# Transcriptome sequencing uncovers a three–long noncoding RNA signature in predicting breast cancer survival

**DOI:** 10.1038/srep27931

**Published:** 2016-06-24

**Authors:** Wenna Guo, Qiang Wang, Yueping Zhan, Xijia Chen, Qi Yu, Jiawei Zhang, Yi Wang, Xin-jian Xu, Liucun Zhu

**Affiliations:** 1 School of Life Sciences, Shanghai University, Shanghai, 200444, P.R. China; 2 State Key Laboratory of Pharmaceutical Biotechnology, School of Life Sciences, Nanjing University, Nanjing, 210093, P.R. China; 3 Department of Radiation Oncology, Fudan University Shanghai Cancer Center; Department of Oncology, Shanghai Medical College, Fudan University, Shanghai, P.R. China; 4 College of Information Science and Technology, Sanda University, Shanghai, 201207, P.R. China; 5 Department of Mathematics, Shanghai University, Shanghai, 200444, P.R. China

## Abstract

Long noncoding RNAs (lncRNAs) play a crucial role in tumorigenesis. The aim of this study is to identify lncRNA signature that can predict breast cancer patient survival. RNA expression data from 1064 patients were downloaded from The Cancer Genome Atlas project. Cox regression, Kaplan–Meier, and receiver operating characteristic (ROC) analyses were performed to construct a model for predicting the overall survival (OS) of patients and evaluate it. A model consisting of three lncRNA genes (*CAT104*, *LINC01234*, and *STXBP5-AS1*) was identified. The Kaplan–Meier analysis and ROC curves proved that the model could predict the prognostic survival with good sensitivity and specificity in both the validation set (AUC = 0.752, 95% confidence intervals (CI): 0.651–0.854) and the microarray dataset (AUC = 0.714, 95%CI: 0.615–0.814). Further study showed the three-lncRNA signature was not only pervasive in different breast cancer stages, subtypes and age groups, but also provides more accurate prognostic information than some widely known biomarkers. The results suggested that RNA-seq transcriptome profiling provides that the three-lncRNA signature is an independent prognostic biomarker, and have clinical significance. In addition, lncRNA, miRNA, and mRNA interaction network indicated lncRNAs may intervene in breast cancer pathogenesis by binding to *miR-190b*, acting as competing endogenous RNAs.

Breast cancer is one of the most frequent cancers among women. Every year, more than 1,300,000 breast cancer cases occur worldwide. Breast cancer accounts for more than 450,000 deaths yearly^1^. Accurately estimating breast cancer patients’ prognosis, choosing effective treatment protocol for high-risk patients, further improving the prognosis of patients, reducing mortality, and prolonging the survival time have a great clinical significance^2^. In current clinical studies, the histopathological features of breast cancer, including tumor size^3^, clinical stage^4^, subtype^5^, lymph node status^6^ are still taken as conventional prognosis factors. However, the evaluation based on these factors is not comprehensive^6^; the prediction which depended on the clinical stage and immunohistochemistry could only predict the situation of a part of patients^7^, and hence the accuracy in determining the tumor size and clinical stage, along with subjectivity, becomes incompetent. Moreover, the risk in individual patients could not be evaluated^8^. With the development of molecular biology techniques, a large number of studies have confirmed that the molecular markers of prognosis in breast cancer can guide clinical individualized treatments and improve the survival time of patients^9^,^10^.

lncRNAs are a class of molecular markers, and their role in cancer, such as aberrant expression, and their involvement in gene regulation, cell differentiation and proliferation, as well as many cancer biology and metastasis, have obtained increasing attention^11^. Recent reports revealed that lncRNAs show a specific expression variation in cancer, indicating they play an important role in biological function in tumor and have a great potential to be regarded as a prognostic biomarker. For instance, *MALAT1* (metastasis-associated lung adenocarcinoma transcript 1) was found to be overexpressed in oral squamous cell carcinoma, and high expression of this lncRNA was related to poor prognosis^12^. *SPRY4-IT1* (*SPRY4* intronic transcript 1) was proved to play a key role in promoting tumorigenesis and predicting prognosis in gastric cancer^13^. *HOTAIR* is a marker for hepatocellular carcinoma progression and tumor recurrence^14^. In addition, *LINC01133*, a novel lncRNA, was found to be upregulated in lung squamous cell cancer, which could predict patient survival^15^. However, the prognostic value of lncRNAs in breast cancer remains poor. So far, only Meng *et al*. have identified four lncRNAs (*U79277*, *AK024118*, *BC040204*, *AK000974*) as an independent predictor for patient survival in breast cancer using four data sets of microarrays^10^. However, the area under receiver operating characteristic curve (AUC) of their model was only 0.603, exhibiting high false positivity and false negativity. Moreover, the model did not show high reproducibility in predicting the survival time for patients in different breast cancer stages and subtypes. In addition, using microarray data to identify lncRNAs had some inevitable shortcomings, such as the chip sequence did not include all lncRNA genes and could only be used for identifying known lncRNAs. Moreover, multiple probes may have corresponded to one lncRNA so that the expression level of lncRNA was unstable, which made it difficult to reflect the real situation of patients.

TCGA is a project supervised by the National Cancer Institute and the National Human Genome Research Institute^16^. TCGA has generated a large number of data for cancer by high-throughput sequencing techniques. The number of samples is regarded as an important factor in cancer survival research; more than 1000 breast cancer patient samples collected worldwide are found in TCGA, which are enough for identifying prognostic markers of breast cancer and eliminate the influence of individual differences. Moreover, RNA expression data were obtained from high-throughput sequencing using the same platform, particularly helpful in identifying new lncRNAs, which could make up for the shortage of microarray analysis^17^.

This study aimed at identifying lncRNAs associated with the survival of breast cancer patients by analyzing high-throughput sequencing data from TCGA and confirming the prognostic value of the identified lncRNAs by the validation set and an independent cohort of patients in Gene Expression Omnibus (GEO) datasets. Besides, whether the identified lncRNA is pervasive in different age, stage and subtype groups was also investigated, and the clinical significance of lncRNAs serving as molecular prognostic markers was further examined.

## Results

### Patient characteristics

All 1064 patients used in this study were clinically and pathologically diagnosed with breast cancer. The clinical stage and histological subtype were determined using TNM staging method and immunohistochemical technique (SP) molecular typing method, respectively^17^. Here breast cancer was graded into stage 1, 2, 3, and 4, and based on the histological subtype, it was classified into estrogen receptor (ER)-negative and ER-positive, progesterone receptor (PR)-negative and PR-positive, human epidermal growth factor receptor 2 (HER2)-negative and HER2-positive, as well as Triple negative breast cancer (TNBC) subgroups. Additionally, the average age and OS time of these 1064 patients were 58.47 years (range, 26–90 years) and 1044 days, respectively. All the statistical information were summarized in Table 1 and Table S1.

### Identification of lncRNAs associated with the OS of patients from the training set

As shown in the workflow (Fig. S1), the training set was firstly analyzed to identify the potential prognostic lncRNAs, and then the validation data set was performed for validation. By performing univariate Cox proportional hazards regression analysis (see Materials and methods) for lncRNA expression data derived from the training set, a total of 11 lncRNAs related to patient OS (*P* ≤ 0.001) were identified (Table S2). Then all possible combinations of these 11 lncRNAs were analyzed and compared by the multivariate Cox regression analysis, and a model consisting of three lncRNAs was identified as the best prognostic model for predicting the OS of patients by repeated comparison analysis. Subsequently, a risk score was calculated based on a linear combination of the expression levels of all these three lncRNAs, weighted by the coefficients derived from the multivariate Cox regression analysis: Risk score = (−0.26 × expression value of *CAT104*) + (0.201 × expression value of *LINC01234*) + (0.514 × expression value of *STXBP5-AS1*). These three lncRNAs were selected as the predictors for survival, and all these three lncRNAs were verified in the NCBI database by ncbi–BLASTN and classified as ncRNAs in this website. The chromosomal positions of these three lncRNAs and related hazard ratios information are shown in Table S3.

### Association of three lncRNAs and patient OS in the training set

**Figure 1:**
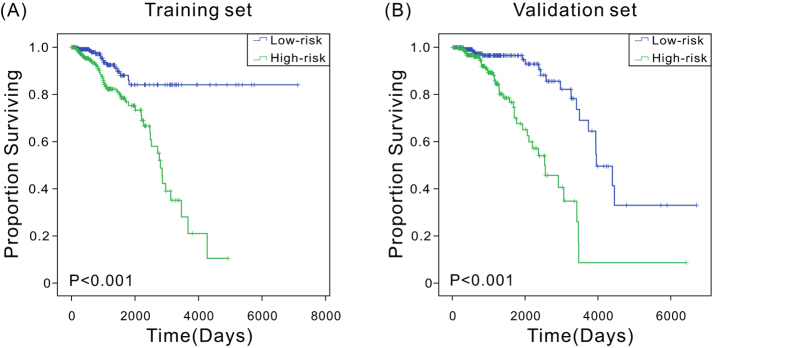
Kaplan–Meier estimates of the overall survival of TCGA patients using the three-lncRNA signature. (**A**) Kaplan–Meier curves for the training-set patients (*n* = 532); (**B**) Kaplan–Meier curves for the validation-set patients (*n* = 532). Two-sided log-rank test was performed to evaluate the survival differences between the two curves.

Hazard ratios (HRs) from the Cox regression analysis showed that the expression levels of the three-lncRNA signature were significantly associated with patient survival (*P* < 0.0001, HR: 2.72, 95%CI: 1.952–3.789). Kaplan–Meier analysis was performed to examine the association between the expression levels of the three-lncRNA signature and patient OS. The patients were divided into low-risk (*n* = 266) and high-risk (*n* = 266) groups using the median risk score as the cutoff point. Clinical characteristics of breast cancer patients belonging to low or high risk group were summarized in Table S4. Compared with the low-risk group, patients in the high-risk group had significantly (*P* < 0.0001) shorter OS (Fig. 1A). In addition, the univariate Cox regression and Kaplan–Meier analyses for each of these three lncRNAs were carried out, which yielded similar results that the high-risk group was correlated to poor OS (Fig. S2A).

### Validation of the three-lncRNA signature in the validation set

To confirm the findings of the present study, additional tests for the predictive effects of these three lncRNAs on the survival of breast cancer in the validation set were carried out. According to the processes in the training set analysis, patients in the validation set were further classified into low-risk and high-risk groups with a median risk score (Table S4), and the Kaplan–Meier analysis was performed to compare the differences in patient survival. In consistent with the results obtained from the training set, patients in the high-risk group had a significantly (*P* < 0.0001) shorter OS than patients in the low-risk group (Fig. 1B). The results suggested that the three-lncRNA signature can effectively predict patient survival. Furthermore, when the univariate Cox regression and Kaplan–Meier analyses were performed in the validation set, and relation between the higher-risk score and shorter OS was also found (Fig. S2B). These results suggested that the three-lncRNA signature is likely to play an essential role in the prognostic prediction of breast cancer.

### Evaluation of the risk score performance by the ROC curve analysis in the validation set

**Figure 2:**
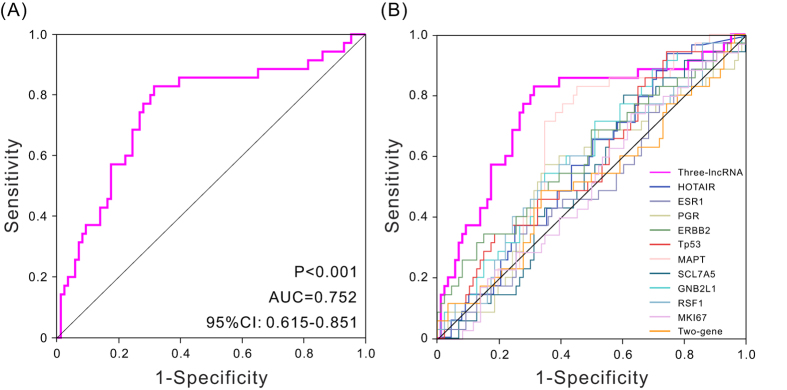
ROC curves analysis show the sensitivity and specificity biomarkers in predicting the patient overall survival. (**A**) ROC curves of the three-lncRNA signature, AUC = 0.752 (*P* < 0.001). (**B**) ROC curves of different biomarkers.

To further assess the predictive accuracy of the three-lncRNA signature, the ROC analysis was performed to evaluate the sensitivity and specificity of survival prediction. The AUC was calculated using 5 years as the cutoff survival time. An AUC of range from 0.7 to 0.9 is considered excellent discrimination^18^. Here the AUC value was 0.752 (*P* < 0.001, 95%CI: 0.651–0.854) (Fig. 2A), indicating the three-lncRNA signature had high sensitivity and specificity, therefore, it can be used to predict the prognostic survival of patients with high accuracy, and it is of great significance in clinic application.

### Comparison of the three-lncRNA signature and known biomarkers

Previous researches have identified several biomarkers as prognostic predictor for breast cancer. For instance, the cell proliferation antigen *MKI67* (also known as *Ki-67*) has often been shown to be a powerful predictor of poor clinical outcome^19^; *HOTAIR* expression serve as an independent biomarker for the prediction of the risk of metastasis in breast cancer patients^20^; *ESR1* (*ER*), *PGR* (*PR*) and *ERBB2* (*HER-2*) were all powerful predictive biomarker^21^. To determine superiority of the three-lncRNA signature, the sensitivity and specificity of 11 known biomarkers were identified in this study (Fig. 2B). The AUC of these biomarkers were shown in Table S5. Comparing with other mentioned biomarkers, the AUC of our three-lncRNA signature was much larger (the AUC of *TP53*, *HOTAIR, ESR1*, *PGR*, and *ERBB2*, *MKI67* were 0.582, 0.580, 0.501, 0.546, 0.599, and 0.509, respectively). These results inspiringly revealed that compared with several applied clinical biomarkers, the three-lncRNA signature was a superior predictor in terms of clinical outcome prediction of breast cancer.

### Validation of the three-lncRNA signature for survival prediction using microarray data

**Figure 3:**
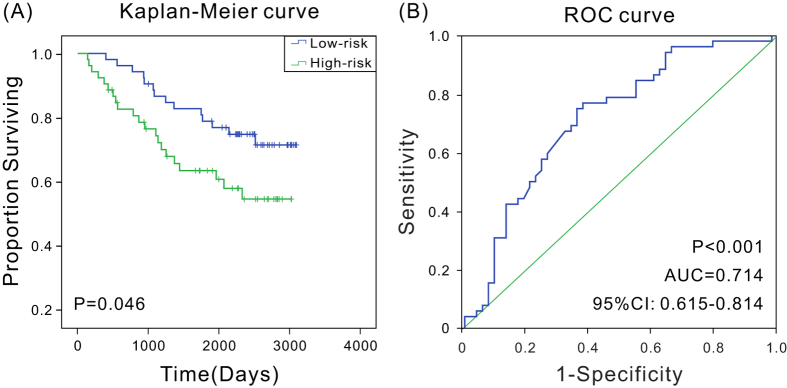
Kaplan–Meier and ROC analysis for overall survival of patients from GSE42568. (**A**) Kaplan–Meier survival curves show correlation between expression of three-lncRNA signature and overall survival of patients; (**B**) ROC curves show the sensitivity and specificity of the three-lncRNA signature in predicting the patient overall survival, AUC = 0.714 (*P* < 0.001).

To evaluate the prognostic values of the three-lncRNA signature in independent cohort of patients from different types of dataset, microarray expression data and corresponding clinical data of 104 breast cancer patients (GSE42568, HG-U133A Plus2 platform)^22^ were downloaded from GEO datasets. Kaplan–Meier analyses proved that patients in the low-risk group had a significantly longer OS than high-risk group (*P* < 0.05) (Fig. 3A), and ROC analysis showed the three-lncRNA signature had high predictive accuracy as well (AUC = 0.714, *P* < 0.001, 95% CI: 0.615–0.814) (Fig. 3B), suggesting that three-lncRNA signature might also could predict the prognostic survival of breast cancer patients in different types of datasets.

### Three-lncRNA signature in different ages, stages and subtypes

Studies have shown that the age at diagnosis, cancer stage and subtype also influence patient prognostic survival^23^. To analyze the clinical effect of the three-lncRNA signature in patients with different ages, patients were divided into two groups based on their ages at initial diagnosis: 565 patients were diagnosed before age 60 (group 1) and 499 patients were diagnosed at or after age 60 (group 2). Using the three-lncRNAs model of this study, the multivariate Cox proportional hazard, Kaplan–Meier, and ROC analyses were performed to further evaluate the effect of risk score on predicting the patient survival time in different age groups, Kaplan–Meier curves show patients in the high-risk group had significantly (*P* < 0.001) shorter OS, and the AUC values were both nearly 0.7 (Fig. S3). The results revealed that the three-lncRNA signature was pervasive in different age patients.

**Figure 4:**
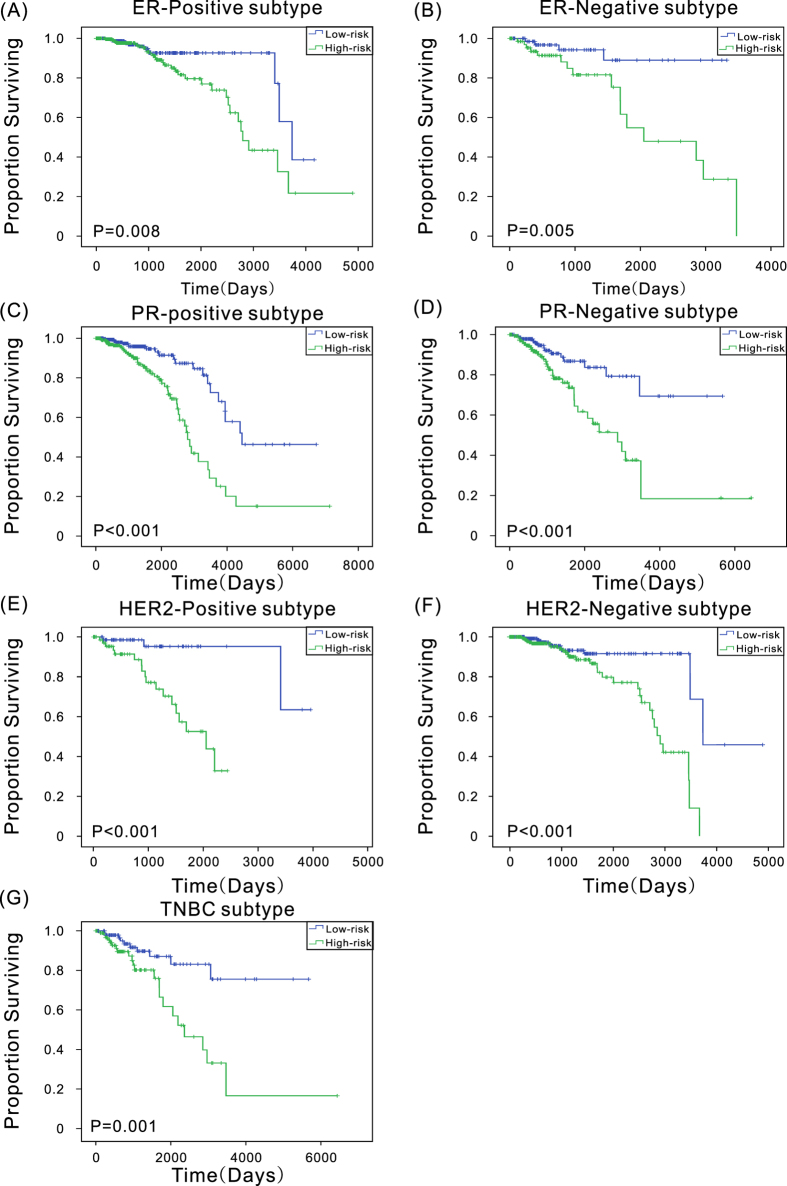
Kaplan–Meier estimates of the overall survival of patients with different breast cancer subtypes.

To investigate whether the three-lncRNA signature was applicable to different breast cancer stages and histology subtypes, the same analyses were conducted in different cancer stages and histology subtypes as in different age groups. In stages 2, 3, and 4, patients in the high-risk group had significantly (*P* < 0.001) shorter OS (Fig. S4), and all the AUC values were more than 0.7 (Fig. S5), suggesting that the three-lncRNA signature has predictive value for different cancer stages. In different subtypes, the differences (*P* < 0.01) in the OS between the two groups were also observed (Fig. 4), and the AUC values in all the subgroup were more than 0.62, especially the AUC value in the ER-positive subgroup was up to 0.82 (Fig. S6), suggesting that the three-lncRNA signature could provide prognostic information in patients with different breast cancer subtypes.

### Independence of the three-lncRNA signature

Considering that the age, clinical stage and subtype might affect patient prognostic survival, we carried out multivariate Cox regression analysis to distinguish whether the three-lncRNA signature could serve as an independent predictor of survival. Stratified analyses were conducted in both training and validation sets, three-lncRNA risk score and age or stage or histology subtype defined as covariates, respectively. The results showed that the three-lncRNA signature, the three-lncRNA signature together with age, or the three-lncRNA signature together with stage, or the three-lncRNA signature together with subtype remained as covariates in the regression model had no significant differences (Table 2), suggesting that the three-lncRNA signature may be used as an independent predictor of patient survival in this study population.

From our previous univariate Cox regression analysis we can see that age, stage, subtype were also significantly related to patient survival (P < 0.05), to further confirm the clinical performance of the three-lncRNA signature, we took the three-lncRNA signature together with age, stage and subtype as biomarker respectively. Multivariate Cox regression analysis were performed to get the combined model in the training set, and Kaplan–Meier and ROC analyses were used to estimate the model in the validation set. The *P*-value in Kaplan–Meier analyses and AUC in ROC analyses were shown in Table S6. These results show all of these biomarkers were not better than the three-lncRNA signature, indicating the three-lncRNA signature could predict the survival well without other clinical performances.

### Identification of miRNA and mRNA associated with these three lncRNAs

**Figure 5:**
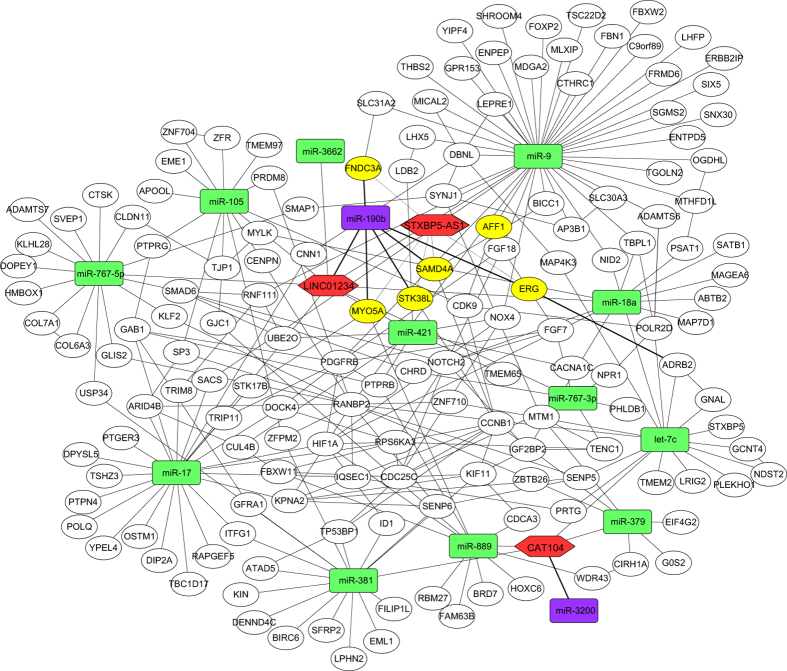
Interaction network of lncRNA, miRNA, and mRNA in breast cancer tumors. Each hexagon (red) indicates lncRNA associated with patient overall survival. Each round rectangle indicates miRNA (purple and green) related to lncRNA, purple indicates miRNA negatively related to lncRNA, and green indicates miRNA positively related to lncRNA. Each ellipse indicates mRNA, yellow indicates targets of miR-190b.

Xia *et al*. have constructed an lncRNA-miRNA-mRNA network to understand the roles of lncRNA in gastric cancer^24^. Here to further investigate the functional mechanisms of these three lncRNAs in breast cancer and evaluate their prognostic values of this disease, a survey of their potential relationships with mRNAs, miRNAs, and lncRNAs was performed using correlation analysis of expression, and 15 miRNAs and 5185 mRNAs were identified to be significantly associated with (*P* < 1e-10) these three lncRNAs. Then the regulatory relationship between these miRNAs and mRNAs as well as the interaction of mRNAs-mRNAs was determined, and the interaction network (Fig. 5) was constructed. Most of the 15 miRNAs were reported to link with breast cancer. For instance, *let-7*, *miR-379*, *miR-381*, and *miR-421* were found to be down-regulated in breast cancer^25^,^26^,^27^,^28^; *miR-17*, *miR-18a*, and *miR-9* turned out to be metastasis-associated^29^,^30^,^31^; and miR-190 was detected to be related to estrogen receptor^32^. In addition, the functional enrichment of target mRNA showed that target mRNAs of these miRNAs were significantly (*P* < 0.01) enriched in the pathway related to cancer, such as pathways in cancer, ubiquitin-mediated proteolysis, and *MAPK* signaling pathway (Table S7). In the present-study network, *miR-190b* and *miR-3200* were negatively related to *LINC01234* and *CAT104*, respectively. And sequence alignment showed that *miR-190b* could map to *LINC01234* from 1339 to 1360, and *miR-3200* could map to *CAT104* from 49 to 70, respectively (Fig. S7). Moreover, some targets of *miR-190b*, such as *ERG*, *STK38L*, and *FNDC3A*, were positively associated with *STXBP5-AS1*, which is in agreement with the functional mechanism reported by Cesana M. *et al*.^33^. The expression of these lncRNAs, miRNAs and mRNAs were showed in Tables S8–10. The results revealed that lncRNAs may intervene in breast cancer pathogenesis by competitively binding to *miR-190b,* acting as competing endogenous RNAs (ceRNAs).

## Discussion

In recent years, with the development of the next-generation sequencing technology, more and more sequencing data were used to study cancer, including cancer diagnosis, therapy, and prognosis^34^. Recent studies primarily focus on the research of ncRNAs associated with cancer. For instance, Hu *et al*. found four miRNAs (*miR-486*, *miR-30d*, *miR-1*, and *miR-499*) that were significantly related to survival of patients with non-small-cell lung cancer by genome-wide serum miRNA expression analysis^35^. And Zou *et al*. detected two novel lncRNAs and their clinical relevance in head and neck cancer pathogenesis by transcriptome sequencing^34^. Baralle *et al*. found *BRCA1* alternative splicing may serves as biomarkers of tumor staging by exome sequencing^36^. The present study is probably the first study using the next-generation sequencing to globally profile the expression of lncRNAs in breast cancer prognostic, and a set of three lncRNAs that can be used to predict the prognosis of breast cancer was determined through the Cox regression and ROC analysis. In this study, 3552 possible lncRNAs were first identified based on MiTranscriptome database, of which 1613 lncRNAs were detected expressing more than half of the total samples in this study. Although 1613 lncRNAs may include several false positive results, these three lncRNAs that served as biomarker were further verified as ncRNAs in the NCBI database. In addition, the results of the univariate Cox regression and Kaplan–Meier analyses for the three individual lncRNAs is not as good as the combination of three lncRNAs both in the training and validation sets, especially for *LINC01234* in validation set, OS of patients in the high-risk group and the low-risk group were different, but they had no significant difference (*P* = 0.103), indicating that although one particular lncRNA may be helpful in predicting patient survival, a combination of three lncRNAs may offer a better potential to fulfill much more sensitive and specific prognostic test.

Breast cancer is clinically heterogeneous because of molecular differences between histologically similar tumors^10^. Many studies have proved that the prognostic outcome of breast cancer patients is diverse for different age, stages and subtypes^17^. Moreover, it is difficult to find a universal prognostic marker. It is clinically significant to find a marker with a better prognostic effect in different breast cancer stages and subtypes. Although the three-lncRNA model could not predict the prognostic outcome of breast cancer accurately in stage 1 due to the lack of samples and low mortality (7.6%), in all other stages, subtypes, and age groups, the model exhibited excellent performance. Patients in high-risk and low-risk groups distinguished by the model have significantly different OS; the AUC value also indicated that the three-lncRNA model has high sensitivity and specificity. Moreover, compared with the three-lncRNA signature, there is no advantage of combining age or stage, or subtype with the three-lncRNA signature. Additionally, the model was also applicative in other dataset, and compared with some famous biomarkers, the three-lncRNA signature could more accurately predict the patient’s outcome. These results suggested that the present-study model was valuable in predicting the survival of patients with different ages, breast cancer stages and subtypes.

Noncoding RNA molecules, especially miRNAs and lncRNAs, are prevalent regulatory factors, which have been shown to play versatile roles in many biological processes^30^,^34^. However, it is still not clear how lncRNAs function in breast cancer. The possible relationships among molecules are crucial to understand the mechanism underlying breast cancer tumorigenesis. For three lncRNAs in this study, *CAT104* is located within the intron of *PDE4DIP*, which has been reported in renal cancer; *STXBP5-AS1* is an antisense RNA, which has been detected differential expressed in lung squamous cancer; *LINC01234* is a long intergenic non-protein coding RNA, which is associated with the survival of patients with oesophageal squamous cell cancer^37^. The functional study of these three lncRNAs identified was not reported in breast cancer so far. Nevertheless, analyses of lncRNA–miRNA and lncRNA–mRNA interactions using gene expression correlation were performed, and interaction network of lncRNA, miRNA, and mRNA were constructed, but why the three lncRNA can server as a prognostic marker? In fact, there is no definitive answer so far. On one hand, many studies have found that lncRNAs acting as ceRNAs influence posttranscriptional regulation by binding to or sequestering specific miRNAs to protect the target mRNAs from repression in both plants and animals^33^,^38^. In this study, *LINC01234* was found to be negatively related to miR-190b, which was similar to *LINC01234* in sequences, and *miR-190b* was reported to be down-regulated in breast cancer and was related to estrogen receptor in several studies. Moreover, *ERG*, a target of *miR-190b*, plays an important role in cell adhesion and cell-cycle regulation, and its expression is differentially related to prognosis in ER-Positive breast cancer^32^. Another target, transcription factor 3 (*TCF3*), was found to participate in the regulation of breast cancer cell differentiation state and tumor formation by repressing Wnt-pathway target genes^39^. In addition, a negative correlation was also observed between *CAT104* and *miR-3200*, and the sequence alignment showed that *CAT104* could bind to *miR-3200*. However, to date, there is no report about the biological role of *miR-3200* in breast cancer. The findings of this study revealed that lncRNAs may be involved in breast cancer by competitively binding to *miR-190b*. On the other hand, there’s also the possibility that the change of expression level of these three lncRNAs are just the result of tumorigenesis and tumor progression of breast cancer. In other word, these three lncRNAs may be the downstream node of some other key node in the network. However, information about the clinical effects of these three lncRNAs in breast cancer is insufficient, and further experimental studies on these lncRNAs may help in verifying the interaction among lncRNA, miRNA, and mRNA, and understanding the functional mechanism and function of three lncRNAs in prognosis of breast cancer.

In conclusion, this study showed that a three-lncRNA signature is associated with patient survival, and may potentially be used as a new independent prognostic marker to predict the OS of patients with breast cancer. More clinical studies on the functional mechanism of the lncRNAs that have not yet been investigated need to be conducted.

## Materials and Methods

### TCGA breast cancer gene expression data

The breast cancer gene expression data (including RNA and miRNA sequencing data) and the corresponding clinical data were obtained from the publicly available TCGA dataset (https://tcga-data.nci.nih.gov/tcga/)^16^, in which gene expression values were normalized to Fragments Per Kilobase of transcript per Million mapped reads (FPKM) values. Up to August 2015, the TCGA included 1097 breast cancer sample information. To analyze the correlation between lncRNA expression signatures and the corresponding overall survival (OS) in breast cancer, only the data including patients with their survival status information were selected. Finally, 1064 RNA-seq and 755 miRNA-seq information were obtained totally. Then, the 1064 cancer samples were randomly and equally divided into training set and validation set. The training set was used to identify gene expression signature, and the other group was used to validate it. The flow chart of the study is described in Fig. S1.

### Identification of lncRNA

To profile the lncRNA expression in the present-study data, the RNA genes downloaded from the TCGA database were compared with the published lncRNAs of human transcriptome from the MiTranscriptome database^40^. Potential lncRNAs were identified when the following three criteria were met: (i) transcriptome sequence was mapped in the corresponding lncRNAs rather than in any protein coding region, and (ii) transcriptome sequence was not protein coding gene in National Center for Biotechnology Information (NCBI) and Ensembl databases, and (iii) transcriptome sequence was expressed in at least half of the breast cancer tissues. In total, 1613 lncRNA candidates were obtained and used for further prediction of lncRNAs related to patient survival. We renamed these lncRNA based on the MiTranscriptome database and NCBI database. In addition, binary logarithm were performed to describe the expression level of RNA-seq data.

### Statistical analysis

The univariate Cox regression analysis was performed to examine the relationship between the lncRNA expression levels in patients and the OS from the training set with an aim to determine which lncRNAs could potentially be of functional significance in breast cancer prognosis. LncRNAs that were significantly related to patient survival were identified (*P* ≤ 0.001)^10^ and then subjected to the multivariate Cox regression analysis. All possible combinations were analyzed to determine the best one, which could be used to construct a risk score formula that would predict survival in the training set. According to the formula, a risk score for each patient was calculated. Using the median score as the cutoff point, patients in the training set were further divided into low-risk group and high-risk group. Differences in the OS between the two groups in both the training and validation sets were estimated and compared by the Kaplan–Meier method with a two-sided log-rank test^41^. ROC curves were used to compare the sensitivity and specificity of the three-lncRNA risk score in the survival prediction^10^. All the data were analyzed using R scripts.

### Construction of interaction network of lncRNAs, miRNAs and mRNAs

A Perl script was used to calculate the correlation coefficient of expression levels. Two genes were considered to be related only when the *P* value of their expression correlation coefficient were less than 1e-10. Subsequently, regulatory relationships between the miRNAs and mRNAs were predicted following a previous report by Guo *et al*.^42^, then the interaction of protein coding genes were downloaded from STRING database (http://string-db.org/)^43^. Finally, the interaction network of lncRNA, miRNA, and mRNA was constructed using the Cytoscape platform.

## Additional Information

**How to cite this article**: Guo, W. *et al*. Transcriptome sequencing uncovers a three-long noncoding RNA signature in predicting breast cancer survival. *Sci. Rep.* **6**, 27931; doi: 10.1038/srep27931 (2016).

## Supplementary Material

Supplementary Information

## Figures and Tables

**Table 1:** Summary of patient demographics and clinic characteristics (n = 1064).

Characteristic		Trainingset(n = 532)	Validationset(n=532)	Total set (n = 1064)	
Age(years)^a^	Mean	58.50	58.44	58.47	
	<60	273	292	565	53.10%
	≥60	259	240	499	46.90%
Sex	Male	6	5	11	17.18%
	Female	526	527	1053	82.82%
Vital Status	Living	470	469	939	88.25%
	Dead	62	63	125	11.75%
Clinical Stage	I	97	87	184	17.60%
	II	310	292	602	57.55%
	III	104	137	241	23.04%
	IV	10	9	19	1.81%
Clinical Subtype	ER-Negative	113	113	226	21.24%
	PR-Negative	171	159	330	31.01%
	HER-Negative	273	275	548	51.50%
OS_time^b^ (Days)	Mean	1048	1039	1044	
	Range	0–7126	0–6719	0–7126	

^a^The age of patient at diagnosis, ^b^Overall survival time.

**Table 2:** Univariate and multivariate Cox regression analyses in the training and validation set.

Variables	Univariate model			Multivariate model		
	HR	95% CI of H	*P*-Value^a^	HR	95% CI of HR	*P*-Value^a^
Training set (N = 532)						
Three-lncRNA risk score	2.72	1.95–3.79	3.31E-09	2.74	1.98–3.81	1.50E-09
Age	1.03	1.10-1.05	4.83E-03	1.03	1.01–1.05	3.50E-04
Validation set (N = 532)						
Three-lncRNA risk score	2.20	1.59–3.04	2.04E-06	1.99	1.44–2.74	3.14E-05
Age	1.03	1.01–1.05	1.10E-02	1.03	1.01–0.05	1.13E-03
Training set (N = 521)						
Three-lncRNA risk score	3.11	2.08–4.44	3.46E-10	3.1	2.17–4.43	5.91E-10
Stage	1.87	1.34–2.60	2.00E-04	1.97	1.36–2.86	3.00E-04
Validation set (N = 525)						
Three-lncRNA risk score	2.45	1.76–3.41	2.04E-07	2.74	1.77–3.55	2.00E-07
Stage	1.71	1.21–2.42	2.00E-04	1.79	1.24–2.58	2.00E-04
Training set (N = 517)						
Three-lncRNA risk score	2.77	1.95–3.94	1.20E-08	2.62	1.83–3.75	1.37E-07
Subtype	0.72	0.54–0.96	2.30E-02	0.84	0.63–1.13	2.40E-02
Validation set (N = 504)						
Three-lncRNA risk score	2.18	1.47–3.22	1.06E-04	2.33	1.55–3.49	4.10E-05
Subtype	0.98	0.72–1.34	9.12E-01	1.20	0.87–1.65	2.69E-01
