# Supplementary material for: Transcriptome sequencing uncovers a three–long noncoding RNA signature in predicting breast cancer survival
Source: Sci Rep. 2016 Jun 24;6:27931. doi: 10.1038/srep27931 (PMC4919625; doi:10.1038/srep27931)
Supplement: Supplementary Information [file srep27931-s1.pdf]

Transcriptome sequencing uncovers a three–long noncoding RNA signature in predicting breast cancer survival

Wenna Guo[1, #], Qiang Wang[2, #], Yueping Zhan[1], Xijia Chen[1], Qi Yu[3], Jiawei Zhang[4],Yi Wang[1], Xin-jian Xu[5], and Liucun Zhu[1,*]

Supplementary Figure and Table

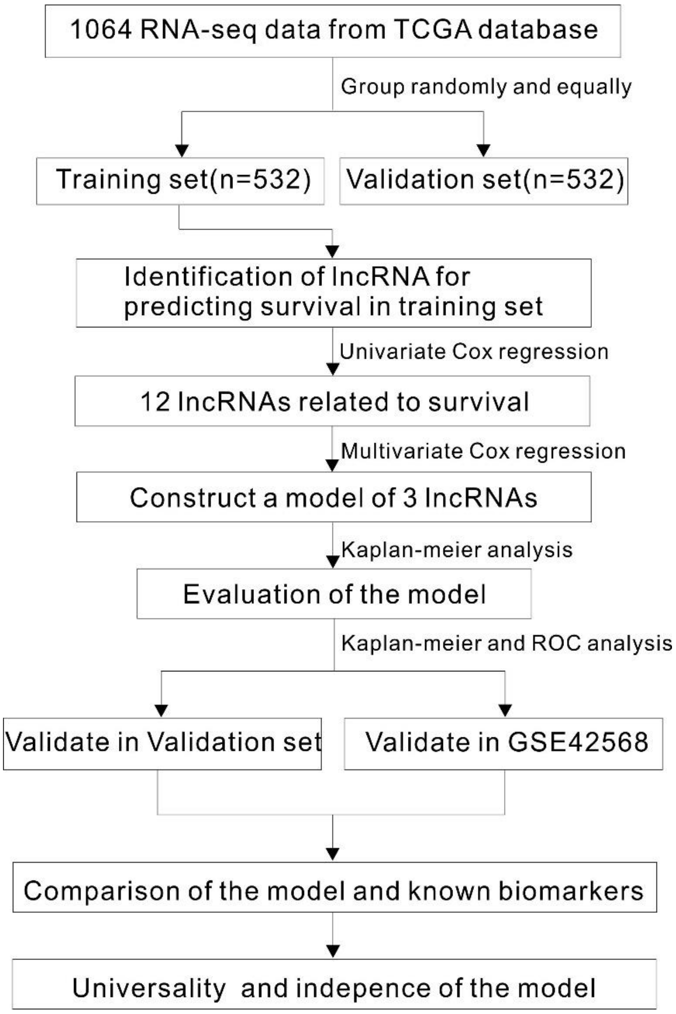

Supplementary Fig S1. Diagram of the study. The process to develop the risk score model and validate the efficiency of the gene signature to predict prognostic outcomes.

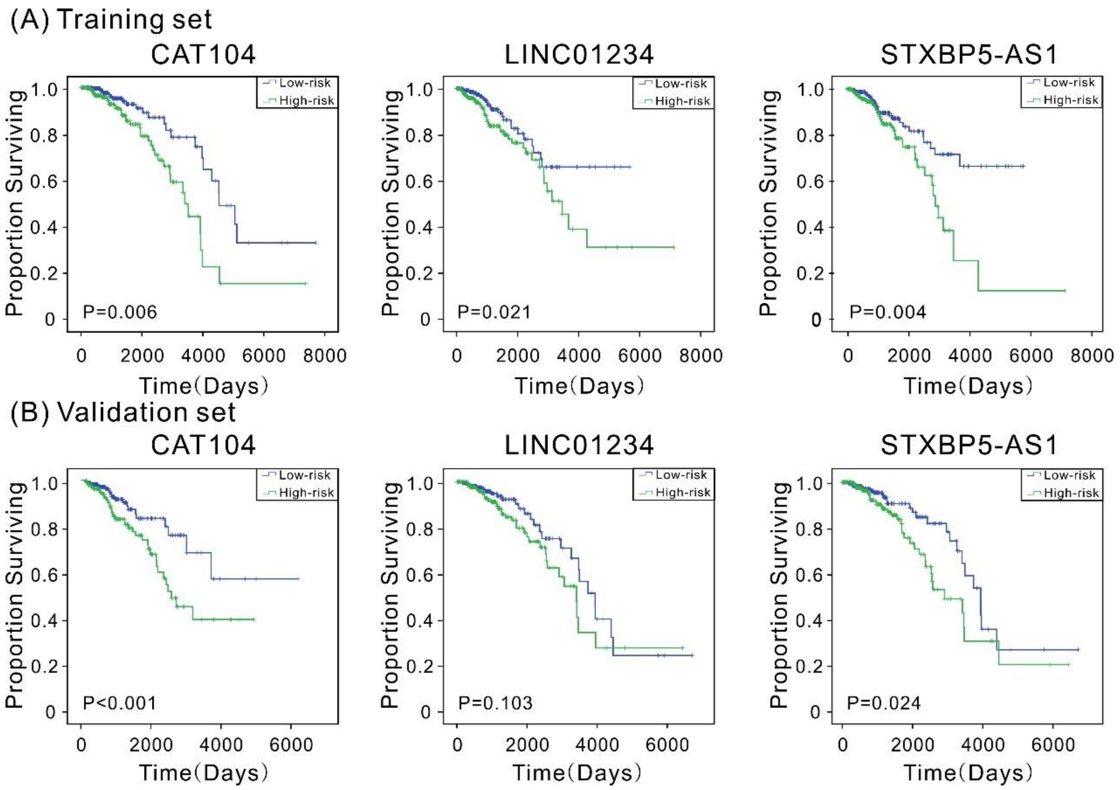

Supplementary Fig S2. Survival information of individual lncRNA in the Training and Validation sets. (A) *CAT104*, *LINC01234*, and *STXBP5-AS1* in the training set. (B) *CAT104*, *LINC01234*, and *STXBP5-AS1* in the Validation set. The survival differences between the two curves were determined by the two-sided log-rank test.

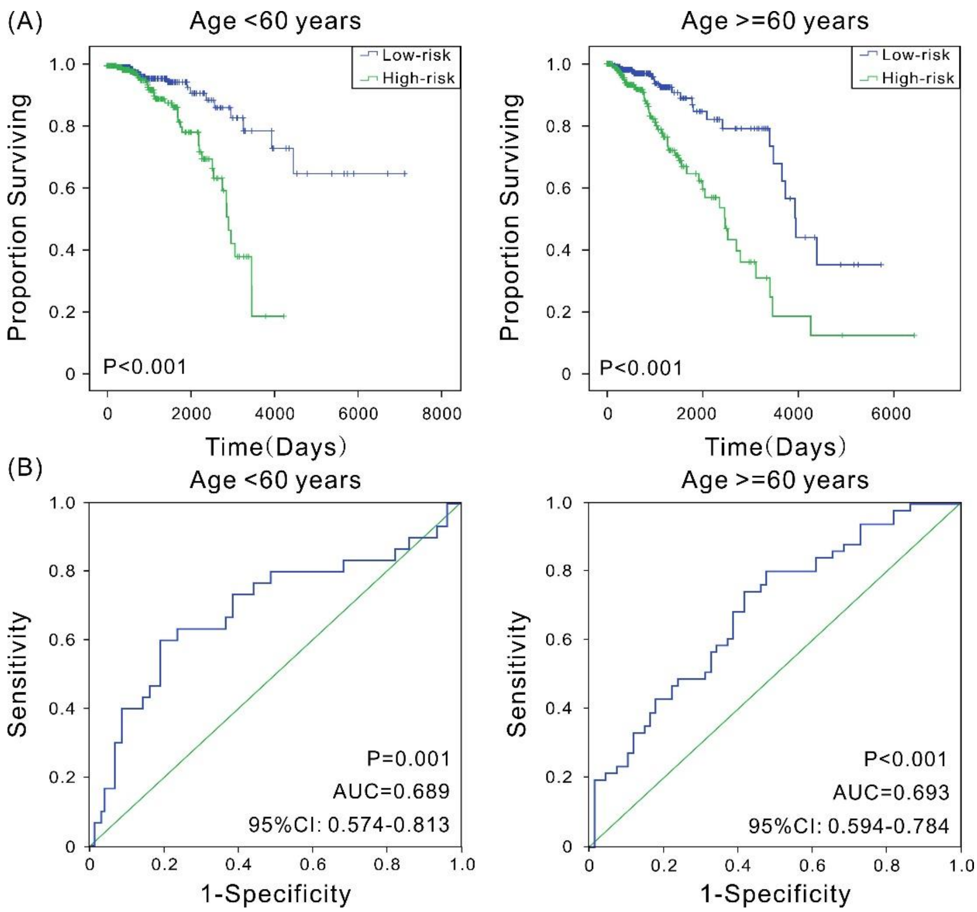

Supplementary Fig S3. Kaplan–Meier and ROC analysis for overall survival of patients in different age groups. (A) Kaplan–Meier survival curves show correlation between expression of three-lncRNA signature and overall survival of patients; (B) ROC curves show the sensitivity and specificity of the three-lncRNA signature in predicting the patient overall survival.

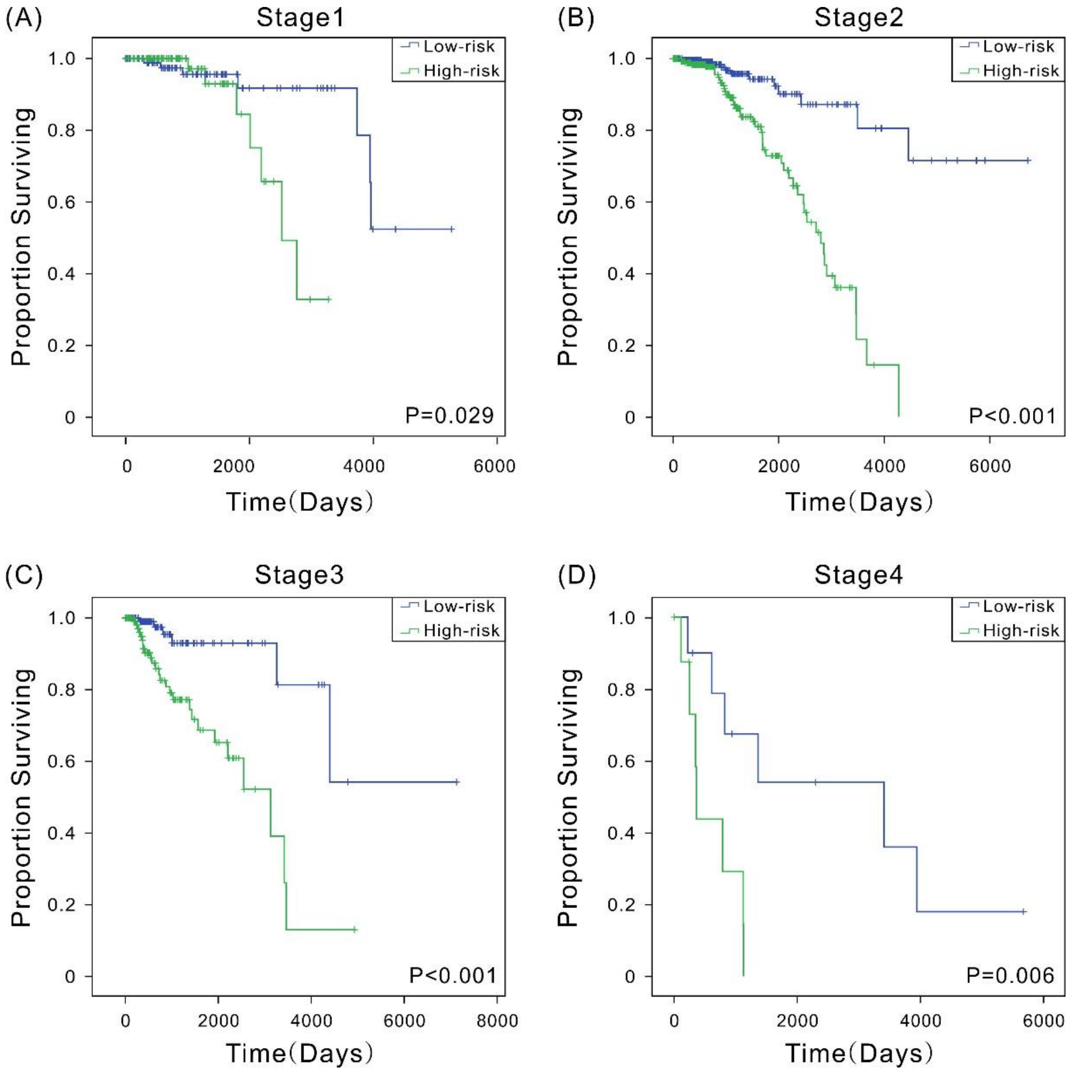

Supplementary Fig S4. Kaplan–Meier estimates of the overall survival of patients with different breast cancer stages. The survival differences between the two curves were determined by the two-sided log-rank test.

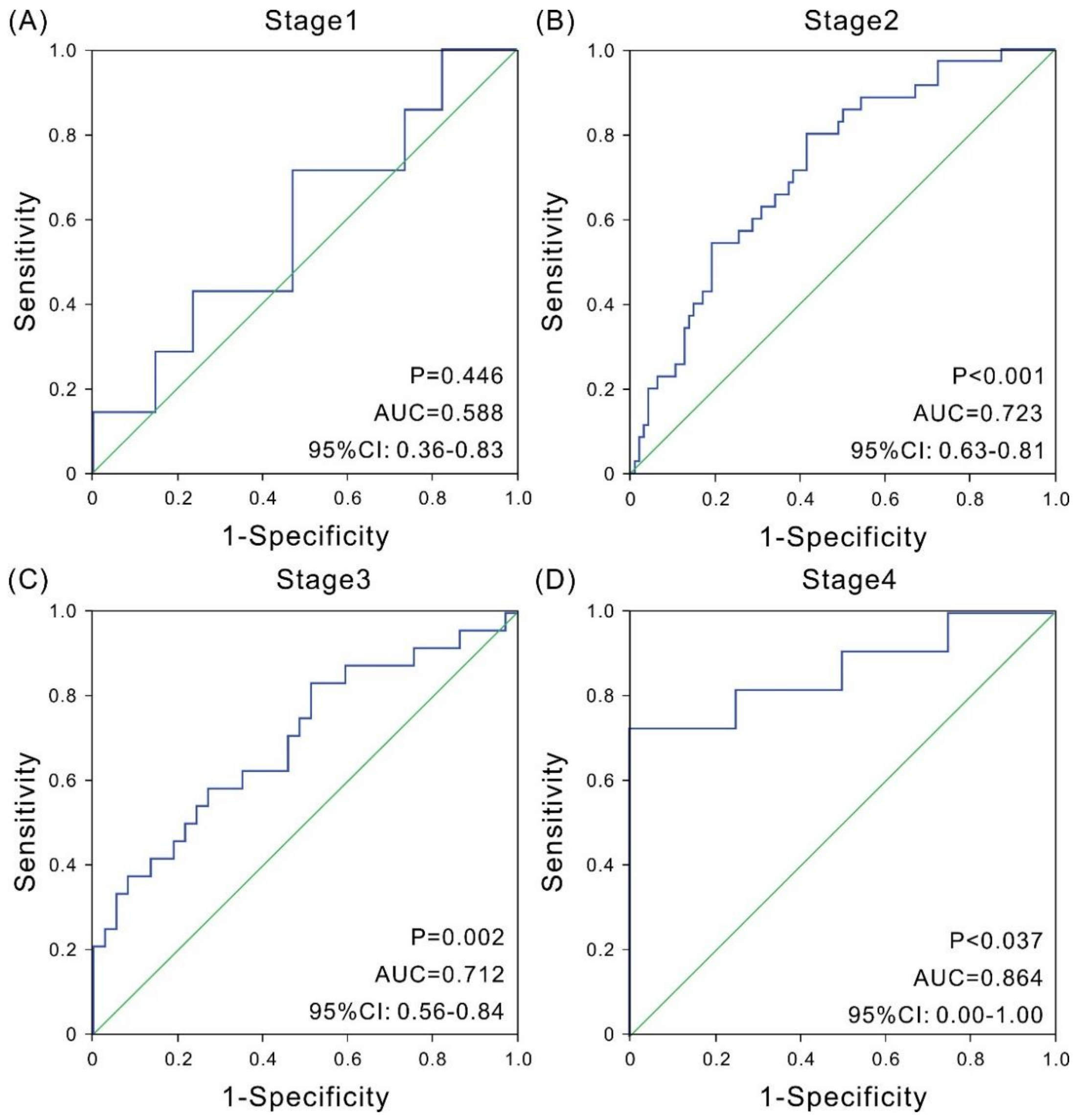

Supplementary Fig S5. ROC analysis of sensitivity and specificity by the three-lncRNA risk score in predicting the overall survival of patients with different breast cancer stages.

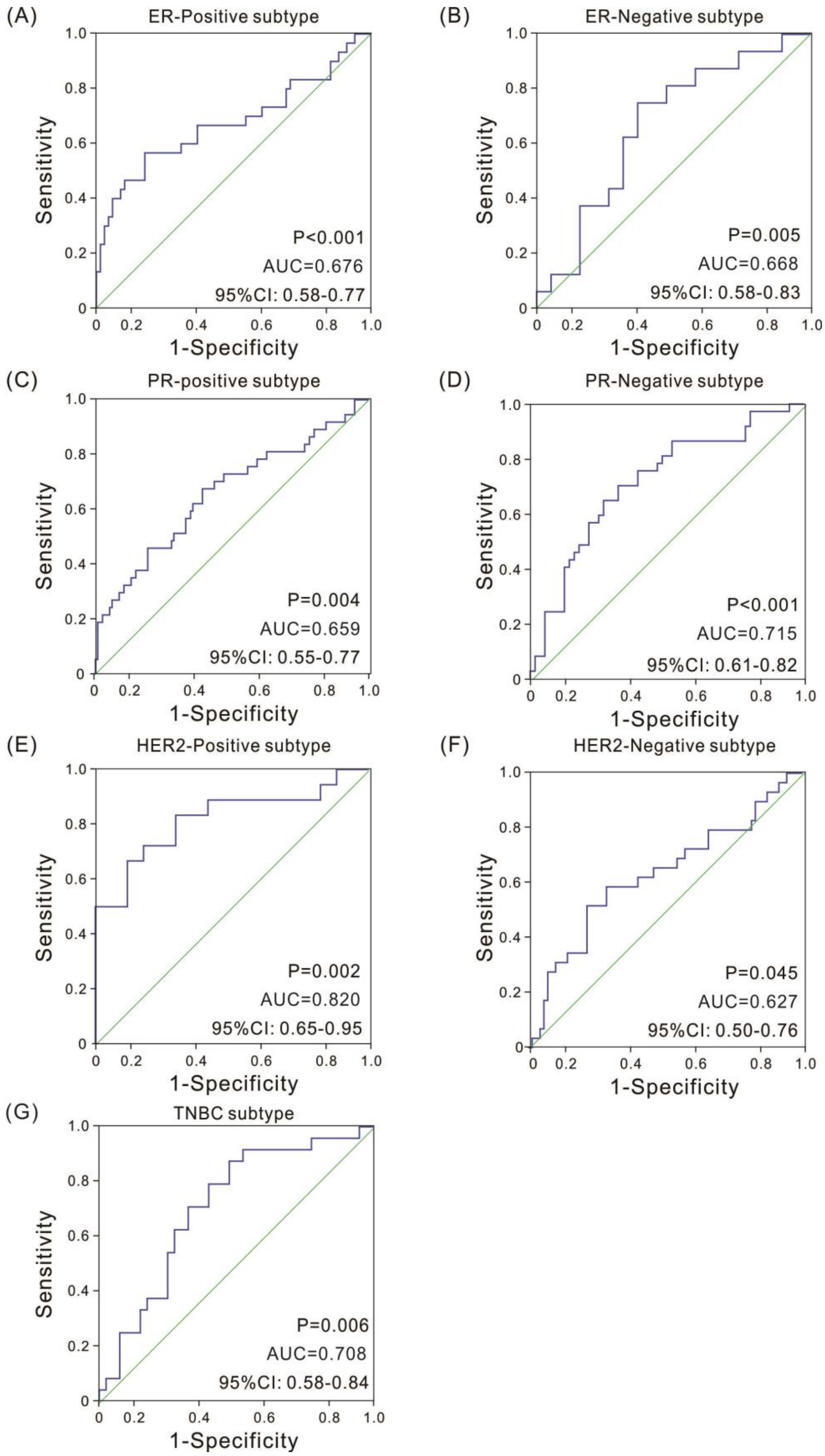

Supplementary Fig S6. ROC analysis of sensitivity and specificity by the three-lncRNA risk score in predicting the overall survival of patients with different breast cancer subtypes.

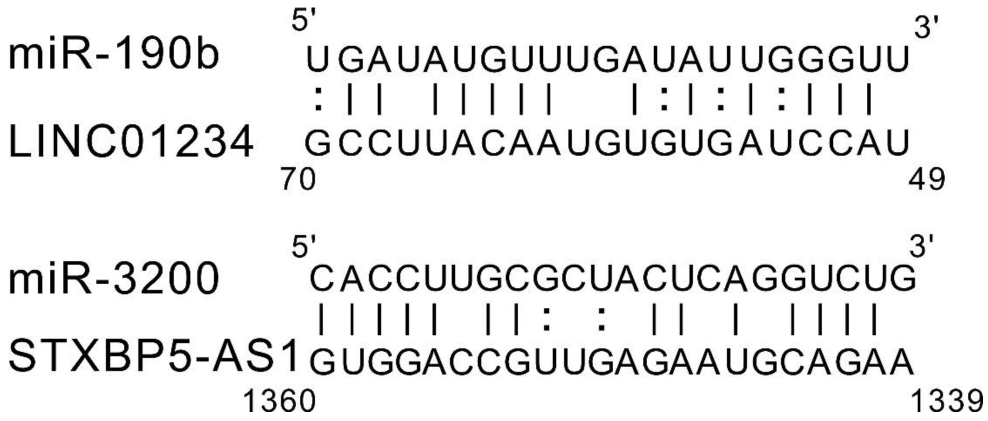

Supplementary Fig S7. Sequence alignment of *miR-190b* and *LINC01234, miR-3200* and *STXBP5-AS1*, respectively.

Supplementary Table S1: Sample list and corresponding information of patients in the training and validation sets

TCGA_ID	OS_status[a]	OS_time	Age[b]	ER Status	PR Status	HER2 Status
Training Set						
TCGA-OL-A5D6	1	1104	71	Negative	Negative	NA
TCGA-E2-A1LH	0	3247	59	Negative	Negative	Negative
TCGA-AO-A0JI	0	1528	56	Positive	Negative	Negative
TCGA-A2-A1FV	0	714	74	Positive	Positive	Negative
TCGA-GM-A2DF	0	1299	53	Negative	Negative	Negative
TCGA-E9-A243	0	519	52	NA	NA	NA
TCGA-AO-A03T	0	1456	42	Positive	Positive	Negative
TCGA-AN-A0XP	0	9	69	Positive	Positive	Positive
TCGA-A8-A06Y	0	791	66	Positive	Positive	Negative
TCGA-AR-A250	0	2707	58	Positive	Negative	Equivocal
TCGA-E2-A159	0	762	50	Negative	Negative	NA
TCGA-D8-A27G	0	409	75	Positive	Positive	Equivocal
TCGA-BH-A0C0	0	1270	62	Positive	Positive	Equivocal
TCGA-B6-A0I2	0	4361	45	NA	NA	NA
TCGA-EW-A1P8	1	239	58	Negative	Negative	Negative
TCGA-BH-A1EX	1	1508	67	Positive	Positive	Positive
TCGA-C8-A26Y	0	13	90	Negative	Negative	Negative
TCGA-AC-A2QI	0	28	76	Positive	Negative	Negative
TCGA-AO-A0J6	0	1140	61	Negative	Negative	Negative
TCGA-D8-A13Z	0	635	51	Negative	Negative	Negative
TCGA-AR-A5QM	0	1665	62	Positive	Positive	Equivocal
TCGA-BH-A0B7	0	1733	42	Positive	Positive	Positive
TCGA-AC-A3BB	0	30	46	Positive	Positive	Negative
TCGA-AQ-A04L	0	3957	48	Positive	Negative	Positive
TCGA-LD-A7W5	0	124	52	Positive	Positive	Negative

TCGA-E2-A14S	0	1009	65	Positive	Positive	Equivocal
TCGA-EW-A1PH	0	607	52	Negative	Negative	Negative
TCGA-C8-A1HM	0	70	74	Positive	Positive	Negative
TCGA-AR-A2LE	0	4356	69	Positive	Negative	NA
TCGA-MS-A51U	0	85	44	Positive	Positive	Negative
TCGA-A8-A08H	0	0	66	Positive	Positive	Positive
TCGA-B6-A402	0	1651	47	Negative	Negative	Negative
TCGA-A7-A0CH	0	1079	79	NA	NA	NA
TCGA-C8-A133	0	0	65	Positive	Positive	NA
TCGA-E9-A22D	0	0	38	Positive	Positive	Positive
TCGA-BH-A0DP	0	476	60	Positive	Positive	Negative
TCGA-A2-A0YM	0	965	67	Negative	Negative	NA
TCGA-E2-A1IJ	0	865	57	Positive	Positive	Equivocal
TCGA-BH-A0EE	0	943	68	Negative	Negative	Positive
TCGA-D8-A1X8	0	783	62	Positive	NA	Negative
TCGA-S3-AA14	0	218	47	Positive	Positive	Positive
TCGA-B6-A0WW	1	558	58	Positive	Positive	NA
TCGA-A2-A4S3	0	666	59	Positive	Positive	Negative
TCGA-B6-A0RN	0	5261	60	Negative	Negative	NA
TCGA-AO-A03L	0	2442	34	Positive	Positive	Positive
TCGA-E9-A1N9	0	399	58	Negative	Positive	Positive
TCGA-A8-A09M	0	1006	75	Positive	Positive	Negative
TCGA-D8-A27P	0	23	64	Positive	Positive	Negative
TCGA-BH-A0H7	0	702	65	Positive	Positive	Negative
TCGA-AR-A1AX	0	1890	64	Positive	Positive	Positive
TCGA-E9-A1N6	0	410	52	Positive	Positive	Positive
TCGA-AR-A24S	0	2976	61	Positive	Positive	Negative
TCGA-A7-A4SF	0	545	54	Positive	Negative	Positive
TCGA-AN-A04C	0	54	51	Negative	Negative	Positive
TCGA-XX-A89A	0	278	68	Positive	Positive	Negative
TCGA-A2-A0D0	0	643	60	Negative	Negative	Negative
TCGA-A2-A0CR	0	3283	54	Positive	Positive	Negative
TCGA-A7-A4SC	0	446	62	Positive	Negative	Equivocal
TCGA-E9-A5UP	0	438	63	NA	NA	NA
TCGA-C8-A12Z	0	12	45	Negative	Negative	Positive
TCGA-E9-A1NE	0	1088	28	Positive	Positive	Negative
TCGA-AN-A0G0	0	16	56	Negative	Negative	Negative
TCGA-E2-A2P6	0	168	77	Positive	Positive	NA
TCGA-A2-A04W	0	1948	50	Negative	Negative	Positive
TCGA-C8-A3M8	0	7	68	Positive	Positive	Positive
TCGA-A8-A08B	0	1156	52	Positive	Negative	Positive
TCGA-A8-A093	0	546	61	Positive	Positive	Negative
TCGA-AN-A0FY	0	10	55	Positive	Positive	Negative
TCGA-B6-A40C	0	2164	51	Positive	Positive	Negative

TCGA-A7-A13G	0	718	79	Positive	Positive	Negative
TCGA-AR-A24W	0	991	55	Positive	Positive	Negative
TCGA-D8-A1JU	0	447	51	Positive	Positive	Negative
TCGA-E2-A140	0	1359	76	Positive	Positive	Equivocal
TCGA-A8-A08R	0	30	52	Negative	Negative	Negative
TCGA-BH-A0DH	0	1156	63	Positive	Positive	Negative
TCGA-BH-A0H5	0	1080	45	Positive	Positive	NA
TCGA-C8-A137	0	125	34	Negative	Negative	Positive
TCGA-A8-A06U	1	883	80	Positive	Positive	Positive
TCGA-OL-A66J	0	1996	80	Positive	Positive	NA
TCGA-A7-A0D9	0	1139	37	Positive	Negative	Negative
TCGA-GM-A2DI	0	1883	52	Negative	Negative	NA
TCGA-LL-A8F5	0	189	61	Positive	Negative	Negative
TCGA-A7-A5ZX	0	336	48	Positive	Positive	Negative
TCGA-GI-A2C8	0	225	63	Positive	Positive	Negative
TCGA-BH-A0BD	0	554	47	Positive	Positive	Negative
TCGA-B6-A0RI	0	7126	44	Positive	Positive	NA
TCGA-AO-A0J2	0	997	41	Negative	Negative	Equivocal
TCGA-A2-A25C	0	523	50	Positive	Positive	Negative
TCGA-A2-A0D4	0	767	37	Positive	Positive	Negative
TCGA-AC-A23H	1	174	90	Positive	Negative	Positive
TCGA-A8-A08X	0	1308	43	Negative	Negative	Positive
TCGA-S3-AA11	0	169	67	Positive	Positive	Equivocal
TCGA-A7-A2KD	0	679	53	Positive	Positive	Positive
TCGA-AR-A5QQ	1	322	68	Negative	Negative	Negative
TCGA-D8-A145	0	410	80	Positive	Positive	Equivocal
TCGA-AO-A124	0	3120	38	Negative	Negative	Negative
TCGA-A2-A0CY	0	1673	63	Positive	Positive	Negative
TCGA-A8-A0AD	0	1157	83	Positive	Positive	Negative
TCGA-B6-A0WX	1	653	40	Negative	Negative	NA
TCGA-AC-A23C	0	29	62	Positive	Positive	Positive
TCGA-BH-A0BP	0	1481	76	Positive	Positive	Negative
TCGA-PE-A5DC	1	1430	72	Positive	Positive	Positive
TCGA-A2-A0SV	1	825	63	Positive	Positive	Equivocal
TCGA-BH-A0W3	0	728	58	Positive	Positive	Equivocal
TCGA-E2-A14P	0	1246	79	Negative	Negative	Positive
TCGA-E2-A10F	0	878	47	Positive	Positive	Equivocal
TCGA-D8-A1JN	0	620	80	Positive	Positive	NA
TCGA-AO-A0JB	0	1542	50	Positive	Positive	Negative
TCGA-E2-A105	0	1308	79	Positive	Positive	Equivocal
TCGA-E9-A3X8	0	478	48	Positive	Positive	Positive
TCGA-BH-A0B4	0	1191	65	Positive	Positive	Equivocal
TCGA-AN-A046	0	10	68	Positive	Positive	Negative
TCGA-A8-A09G	0	0	79	Positive	Negative	Positive

TCGA-AR-A0TP	0	3386	43	Positive	Negative	Negative
TCGA-C8-A26Z	0	252	59	Positive	Positive	Negative
TCGA-AO-A03O	1	2483	69	Positive	Positive	Negative
TCGA-OL-A6VO	0	858	43	Negative	Negative	Negative
TCGA-GM-A2DK	0	1896	58	Positive	Negative	Negative
TCGA-E9-A2JS	1	904	72	NA	NA	NA
TCGA-EW-A424	0	715	51	Positive	Positive	Positive
TCGA-A7-A3J0	0	313	62	Positive	Positive	Negative
TCGA-E2-A156	0	726	61	Positive	Positive	Negative
TCGA-BH-A1FB	1	3669	60	Positive	Positive	Negative
TCGA-A7-A0DA	0	1085	62	Negative	Negative	Negative
TCGA-A8-A085	0	1124	44	Positive	Positive	Negative
TCGA-E9-A1R4	0	186	66	NA	NA	NA
TCGA-AR-A0U0	0	1988	73	Negative	Negative	NA
TCGA-E2-A576	0	578	69	Positive	Positive	Equivocal
TCGA-AN-A0XW	0	170	36	Positive	Positive	Positive
TCGA-E2-A10C	0	1220	54	Positive	Positive	Negative
TCGA-AR-A0TQ	0	2240	27	Positive	Negative	Equivocal
TCGA-BH-A0RX	0	170	59	Negative	Negative	Negative
TCGA-BH-A0AW	0	622	56	Positive	Negative	Positive
TCGA-C8-A1HJ	0	5	53	Negative	Negative	Negative
TCGA-AC-A23G	0	330	76	Positive	Positive	Positive
TCGA-B6-A0I6	1	997	49	Negative	Negative	NA
TCGA-EW-A1P7	0	915	59	Negative	Negative	Equivocal
TCGA-W8-A86G	0	161	66	Positive	Positive	Negative
TCGA-B6-A0RP	1	3126	73	Positive	Positive	NA
TCGA-A8-A07E	0	608	81	Positive	Positive	Negative
TCGA-E2-A1IU	0	337	60	Positive	Positive	Negative
TCGA-AR-A24H	0	4894	65	Positive	Positive	Negative
TCGA-AC-A62Y	0	530	79	Positive	Positive	Negative
TCGA-C8-A26X	0	11	58	Negative	Negative	Negative
TCGA-A2-A0CU	1	158	73	Positive	Positive	Equivocal
TCGA-AC-A62X	0	34	72	Positive	Negative	NA
TCGA-A8-A08G	0	607	41	Positive	Positive	Positive
TCGA-C8-A134	0	34	52	Negative	Negative	Equivocal
TCGA-A2-A0SY	0	1347	62	Positive	Positive	NA
TCGA-LL-A73Z	1	227	55	Positive	Positive	Equivocal
TCGA-A2-A0EO	0	2442	54	Positive	Positive	Negative
TCGA-A2-A0EX	0	752	46	Positive	Positive	Negative
TCGA-E9-A1R5	0	42	63	NA	NA	NA
TCGA-OL-A5RV	0	1062	43	Positive	Positive	NA
TCGA-E2-A15L	0	626	65	Positive	Positive	Equivocal
TCGA-BH-A18M	1	2207	39	Positive	Positive	Positive
TCGA-D8-A27F	0	488	40	Negative	Negative	Negative

TCGA-D8-A147	0	584	45	Negative	Negative	Negative
TCGA-A8-A076	0	1642	66	Positive	Positive	Positive
TCGA-BH-A0HW	0	1561	62	Positive	Negative	Equivocal
TCGA-AR-A2LH	1	616	55	Negative	Negative	Equivocal
TCGA-OL-A66N	0	792	59	Positive	Negative	NA
TCGA-BH-A18V	1	1556	48	Negative	Negative	Negative
TCGA-E2-A1IN	0	675	60	Positive	Positive	Negative
TCGA-A2-A0T4	0	624	62	Positive	Positive	Equivocal
TCGA-AR-A24Q	0	3172	49	Positive	Negative	Negative
TCGA-B6-A0X7	1	1791	62	Positive	Positive	NA
TCGA-BH-A0H0	0	461	69	Positive	Positive	Negative
TCGA-A2-A25E	0	2796	34	Positive	Positive	Equivocal
TCGA-AN-A0FL	0	231	62	Negative	Negative	Positive
TCGA-A7-A0CG	0	1043	78	Positive	Negative	Negative
TCGA-E9-A1RG	0	647	62	NA	NA	NA
TCGA-D8-A13Y	0	362	52	Positive	Positive	Negative
TCGA-A2-A04P	1	548	36	Negative	Negative	NA
TCGA-A8-A07I	0	426	69	Positive	Negative	Positive
TCGA-LL-A5YO	0	440	50	Negative	Negative	Negative
TCGA-C8-A138	0	114	54	Positive	Negative	Equivocal
TCGA-E2-A14T	0	1013	52	Positive	Positive	Negative
TCGA-E2-A1IK	0	689	71	Positive	Positive	Negative
TCGA-C8-A12X	0	385	62	Positive	Positive	Negative
TCGA-A2-A0EP	0	3273	56	Positive	Negative	Negative
TCGA-E9-A244	0	21	54	NA	NA	NA
TCGA-OL-A66P	0	428	75	Negative	Negative	NA
TCGA-GI-A2C9	0	3342	58	Negative	Negative	Negative
TCGA-BH-A0BV	0	1519	78	Positive	Positive	Negative
TCGA-AR-A0U3	0	3468	59	Positive	Positive	Negative
TCGA-BH-A18K	1	2763	46	Positive	Positive	Negative
TCGA-E2-A570	0	931	47	Positive	Positive	Equivocal
TCGA-D8-A143	0	431	51	Negative	Negative	Negative
TCGA-LL-A5YP	0	450	49	Positive	Negative	Negative
TCGA-AO-A12A	0	2755	47	Positive	Positive	Negative
TCGA-BH-A42T	0	30	75	Positive	Positive	Positive
TCGA-B6-A0RH	0	5749	51	Positive	Positive	NA
TCGA-OL-A5RU	0	1219	63	Positive	Positive	NA
TCGA-BH-A1F6	1	2965	51	Negative	Negative	Negative
TCGA-AC-A2FM	1	792	87	NA	NA	NA
TCGA-GM-A3XN	0	2019	44	Positive	Positive	Negative
TCGA-A8-A091	0	1004	61	Positive	Negative	Negative
TCGA-A8-A094	0	0	75	Positive	Negative	Negative
TCGA-AN-A0AM	0	5	56	Positive	Negative	Negative
TCGA-AC-A6NO	0	51	43	Positive	Positive	Negative

TCGA-E2-A572	0	831	72	Positive	Positive	Equivocal
TCGA-B6-A0RQ	1	4273	68	Positive	Positive	NA
TCGA-A2-A0CQ	0	2695	62	Positive	Positive	Equivocal
TCGA-C8-A1HI	0	343	40	Positive	Positive	Negative
TCGA-BH-A0B2	0	1242	43	Positive	Positive	Negative
TCGA-AN-A0FJ	0	242	59	Positive	Negative	Positive
TCGA-A8-A07O	0	304	51	Negative	Negative	Negative
TCGA-E9-A1RH	0	1052	63	NA	NA	NA
TCGA-GM-A3XG	0	1330	46	Positive	Positive	Negative
TCGA-A8-A06N	0	0	66	Positive	Negative	Negative
TCGA-AR-A24K	0	1548	46	Positive	Positive	Equivocal
TCGA-A2-A0CL	0	1964	37	Positive	Positive	Negative
TCGA-AR-A1AU	0	2318	39	Positive	Positive	Equivocal
TCGA-AR-A2LR	0	620	49	Negative	Negative	Negative
TCGA-A7-A5ZW	0	326	47	Positive	Positive	Negative
TCGA-A8-A086	0	396	59	Positive	Positive	Negative
TCGA-E9-A2JT	0	23	63	NA	NA	NA
TCGA-E9-A1NI	0	300	51	Positive	Positive	Negative
TCGA-BH-A1EO	1	2798	68	Positive	Positive	Negative
TCGA-A1-A0SF	0	1463	54	Positive	Positive	Negative
TCGA-D8-A1X7	0	509	40	Positive	Positive	Negative
TCGA-AO-A03U	1	1793	31	Negative	Negative	Negative
TCGA-AO-A1KQ	0	1882	84	Positive	Positive	Negative
TCGA-E9-A295	0	22	71	Positive	Positive	Positive
TCGA-A7-A0CE	0	1074	57	Negative	Negative	Equivocal
TCGA-A2-A1G6	0	501	50	Negative	Negative	Negative
TCGA-E2-A15T	0	456	65	Positive	Positive	Equivocal
TCGA-A8-A0A2	0	579	66	Positive	Positive	Negative
TCGA-LD-A9QF	0	266	73	Negative	Negative	Positive
TCGA-BH-A0BO	0	1428	56	Positive	Positive	Negative
TCGA-BH-A0DO	0	525	78	Positive	Positive	Negative
TCGA-A2-A0EM	0	3094	73	Positive	Positive	NA
TCGA-AO-A0JC	0	1547	64	Positive	Positive	Negative
TCGA-AR-A24V	0	2596	52	Positive	Positive	Negative
TCGA-EW-A2FR	0	1673	59	Negative	Negative	Equivocal
TCGA-A8-A095	0	1277	45	Positive	Positive	Negative
TCGA-E9-A54X	0	375	85	Positive	Positive	Negative
TCGA-GM-A2DM	0	2331	57	Positive	Positive	Negative
TCGA-EW-A1OV	0	789	56	Negative	Negative	Negative
TCGA-EW-A2FS	0	1604	41	Positive	Negative	Negative
TCGA-LL-A440	0	759	61	Positive	Positive	Equivocal
TCGA-GM-A2D9	1	1812	69	Positive	Positive	NA
TCGA-A2-A0YE	0	554	48	Negative	Negative	Negative
TCGA-E9-A1R3	0	64	70	NA	NA	NA

TCGA-A2-A0SX	0	1534	48	Negative	Negative	Negative
TCGA-BH-A5J0	0	715	63	Positive	Positive	Negative
TCGA-A8-A0A4	0	396	73	Positive	Positive	Negative
TCGA-AN-A0XO	0	375	59	Positive	Negative	Negative
TCGA-AR-A0TX	0	1972	64	Positive	Positive	Positive
TCGA-BH-A18I	0	220	53	Positive	Positive	Positive
TCGA-BH-A0E7	0	1363	79	Positive	Positive	Negative
TCGA-A2-A1G0	0	616	49	Positive	Positive	Equivocal
TCGA-A2-A0EW	0	905	53	Positive	Positive	Negative
TCGA-E9-A1NA	0	1112	58	Positive	Positive	Positive
TCGA-D8-A1XV	0	461	84	Positive	Positive	Equivocal
TCGA-BH-A0BR	0	1633	59	Positive	Positive	Negative
TCGA-D8-A73X	0	368	53	Positive	Positive	Negative
TCGA-B6-A0IJ	0	5383	42	Positive	Positive	NA
TCGA-D8-A27R	0	307	41	Positive	Positive	Equivocal
TCGA-BH-A1F5	1	2712	62	Positive	Positive	Negative
TCGA-A2-A1G1	0	584	85	Negative	Negative	Equivocal
TCGA-C8-A1HE	0	375	59	Positive	Positive	Equivocal
TCGA-BH-A5IZ	0	567	51	Positive	Negative	Negative
TCGA-E9-A22B	0	692	71	Positive	Negative	Negative
TCGA-E9-A1QZ	0	362	61	NA	NA	NA
TCGA-EW-A1P3	0	1611	48	Positive	Positive	Negative
TCGA-BH-A0W4	0	759	46	Positive	Positive	Negative
TCGA-BH-A8FZ	0	574	58	Positive	Positive	Equivocal
TCGA-AN-A0AK	0	224	76	Positive	Negative	Positive
TCGA-A2-A0CS	0	2298	73	Positive	Positive	Equivocal
TCGA-OL-A66I	0	714	36	Negative	Negative	NA
TCGA-E2-A1LG	0	574	50	Negative	Negative	Equivocal
TCGA-PL-A8LX	0	5	35	NA	NA	NA
TCGA-BH-A1FD	1	1009	68	Positive	Positive	Negative
TCGA-OL-A5RZ	0	679	57	Positive	Negative	NA
TCGA-E2-A1L6	0	1648	44	Positive	Positive	Negative
TCGA-A7-A26F	0	738	55	Negative	Negative	Equivocal
TCGA-EW-A1IZ	0	554	53	Positive	Positive	Negative
TCGA-D8-A1XR	0	482	56	Positive	Positive	Negative
TCGA-A8-A07S	0	243	73	Positive	Negative	Negative
TCGA-AO-A0JM	0	2184	40	Positive	Positive	Positive
TCGA-B6-A0RL	1	2469	60	Positive	Positive	NA
TCGA-S3-AA0Z	0	511	63	Positive	Positive	Equivocal
TCGA-AR-A1AM	0	2315	52	Positive	Positive	Equivocal
TCGA-E9-A1R7	0	645	64	NA	NA	NA
TCGA-D8-A1X9	0	727	66	Positive	Positive	NA
TCGA-C8-A12N	0	358	58	Positive	Positive	Negative
TCGA-AN-A0FS	0	191	55	Positive	Negative	Positive

TCGA-A2-A0YD	0	769	63	Positive	Positive	NA
TCGA-OL-A66L	0	1301	71	Positive	Positive	NA
TCGA-E9-A5FL	0	24	65	Negative	Negative	Negative
TCGA-A8-A082	0	549	58	Positive	Positive	Negative
TCGA-A8-A0A1	0	365	84	Positive	Positive	Negative
TCGA-BH-A0DL	0	1467	64	Positive	Negative	Negative
TCGA-A2-A4S1	0	820	66	Positive	Negative	Negative
TCGA-BH-A0BC	0	974	60	Positive	Positive	Negative
TCGA-B6-A0IC	1	1542	90	Positive	Positive	NA
TCGA-BH-A0B6	0	1732	47	Positive	Positive	Positive
TCGA-C8-A130	0	96	52	Positive	Positive	Equivocal
TCGA-BH-A0HO	0	76	48	Positive	Positive	Negative
TCGA-A1-A0SD	0	437	59	Positive	Positive	Negative
TCGA-BH-A0DI	0	673	63	Positive	Positive	Negative
TCGA-A8-A09X	1	426	62	Negative	Negative	Negative
TCGA-A8-A08Z	0	1217	76	Positive	Positive	Negative
TCGA-A1-A0SH	0	1437	39	Negative	Positive	Equivocal
TCGA-A2-A0T0	0	533	59	Negative	Negative	Negative
TCGA-A8-A09I	0	1371	84	Positive	Positive	Positive
TCGA-B6-A0RO	0	4929	71	Positive	Positive	NA
TCGA-BH-A2L8	0	612	45	Positive	Positive	Negative
TCGA-GM-A2DB	0	1616	62	Negative	Negative	Negative
TCGA-E9-A1RB	1	976	40	NA	NA	NA
TCGA-D8-A140	0	403	62	Positive	Positive	Positive
TCGA-A7-A13E	1	614	62	Positive	Negative	Equivocal
TCGA-E2-A1IG	0	997	45	Positive	Positive	Negative
TCGA-A2-A3XW	0	1505	42	Positive	Negative	Negative
TCGA-E9-A3QA	0	918	33	NA	NA	NA
TCGA-BH-A0BF	0	782	56	Positive	Positive	Negative
TCGA-BH-A0H6	0	747	82	Positive	Positive	Negative
TCGA-D8-A1XG	0	448	86	Positive	Negative	Negative
TCGA-AC-A23E	0	72	50	Positive	Positive	NA
TCGA-AR-A24P	0	84	47	Positive	Positive	Equivocal
TCGA-LL-A50Y	0	762	84	Positive	Positive	Equivocal
TCGA-AC-A2FB	0	1234	65	Positive	Positive	Positive
TCGA-A7-A3IZ	0	322	62	Positive	Negative	Equivocal
TCGA-AO-A0J8	0	680	61	Positive	Positive	Negative
TCGA-EW-A1P1	0	1210	68	Negative	Negative	Equivocal
TCGA-E9-A1ND	0	383	75	Negative	Negative	Positive
TCGA-BH-A1ET	1	2520	55	Positive	Positive	Negative
TCGA-E9-A226	0	1038	45	NA	NA	NA
TCGA-OL-A5S0	0	620	66	Positive	Negative	NA
TCGA-AO-A126	0	3307	39	Positive	Positive	Negative
TCGA-E9-A22A	0	1189	74	Positive	Negative	Negative

TCGA-A8-A06X	1	943	77	Positive	Negative	Positive
TCGA-A2-A4RX	0	742	67	Positive	Positive	Negative
TCGA-E2-A1LL	0	1309	73	Negative	Negative	Negative
TCGA-A8-A0A7	0	30	57	Negative	Negative	Positive
TCGA-EW-A423	0	309	75	Positive	Positive	Negative
TCGA-B6-A0IP	0	3374	74	Positive	Positive	NA
TCGA-B6-A0X1	0	5672	48	Negative	Negative	NA
TCGA-A8-A09T	0	579	68	Positive	Positive	Negative
TCGA-E2-A10B	0	1141	67	Positive	Positive	Equivocal
TCGA-E9-A1NH	0	0	71	Positive	Positive	Negative
TCGA-BH-A1FN	1	2192	34	Positive	Positive	NA
TCGA-EW-A1P5	0	703	77	Positive	Positive	Negative
TCGA-BH-A1FH	1	1123	47	Positive	Negative	NA
TCGA-UU-A93S	1	116	63	Negative	Negative	Positive
TCGA-BH-A0B9	0	1572	44	Negative	Negative	Negative
TCGA-WT-AB44	0	791	77	Positive	Positive	Negative
TCGA-E2-A1IL	0	118	78	Positive	Positive	Negative
TCGA-A8-A07G	0	577	65	Positive	Positive	Negative
TCGA-A2-A0YC	0	990	59	Positive	Positive	NA
TCGA-S3-A6ZG	0	207	71	Positive	Positive	Negative
TCGA-BH-A1FE	1	2273	31	Positive	Positive	NA
TCGA-E2-A1L7	0	834	40	Negative	Negative	Negative
TCGA-BH-A0BO	0	1085	54	Positive	Positive	Negative
TCGA-B6-A408	0	2072	55	Positive	Positive	Equivocal
TCGA-BH-AB28	0	185	53	Positive	Positive	Negative
TCGA-C8-A1HF	0	332	48	Negative	Positive	Positive
TCGA-AN-A0AJ	0	244	79	Positive	Positive	Positive
TCGA-E2-A155	0	640	58	Positive	Negative	Negative
TCGA-BH-A1F8	1	749	90	Positive	Positive	Positive
TCGA-A2-A0CT	0	2289	71	Positive	Negative	Equivocal
TCGA-C8-A1HK	0	366	53	Negative	Negative	Positive
TCGA-D8-A27K	0	287	47	Positive	Positive	Negative
TCGA-C8-A1HO	0	0	34	Positive	Positive	Negative
TCGA-LL-A5YM	0	466	88	Positive	Positive	Equivocal
TCGA-GM-A2DO	0	1623	54	Positive	Positive	Negative
TCGA-D8-A1Y2	0	433	71	Positive	Positive	NA
TCGA-A2-A3XU	1	912	35	Negative	Negative	NA
TCGA-A7-A13H	0	899	61	Positive	Positive	Equivocal
TCGA-GM-A4E0	0	2191	67	Positive	Positive	NA
TCGA-AO-A0JE	0	2335	53	Negative	Negative	Positive
TCGA-C8-A12L	0	363	67	Negative	Negative	Equivocal
TCGA-AC-A3YI	0	707	74	Positive	Positive	Positive
TCGA-B6-A0X4	1	860	62	Positive	Positive	NA
TCGA-AO-A0J7	0	618	71	Positive	Positive	Negative

TCGA-BH-A0EA	1	993	72	Positive	Positive	Negative
TCGA-E9-A1R0	0	860	58	NA	NA	NA
TCGA-EW-A1IY	0	258	38	Positive	Positive	Negative
TCGA-A8-A09C	0	31	69	Positive	Positive	Negative
TCGA-AR-A1AQ	0	1310	49	Negative	Negative	Equivocal
TCGA-EW-A1OY	0	908	63	Positive	Positive	Negative
TCGA-BH-A202	0	22	60	Positive	Positive	Positive
TCGA-D8-A1JK	0	612	90	Negative	Positive	Negative
TCGA-BH-A18S	1	2009	79	Positive	Positive	Negative
TCGA-A1-A0SB	0	259	70	Positive	Negative	Negative
TCGA-A7-A3J1	0	343	63	Positive	Positive	Negative
TCGA-AR-A1AY	0	1026	65	Negative	Negative	Negative
TCGA-AN-A0XL	0	10	61	Positive	Positive	Negative
TCGA-A2-A3XS	1	1032	62	Negative	Negative	NA
TCGA-GM-A2DN	0	2352	58	Positive	Positive	Negative
TCGA-A2-A0T5	0	531	39	Positive	Positive	Negative
TCGA-E2-A1IH	0	1026	80	Positive	Positive	Equivocal
TCGA-AQ-A7U7	0	304	55	Positive	Positive	Negative
TCGA-EW-A1PA	0	575	59	Positive	Positive	Negative
TCGA-EW-A1J6	0	875	70	Positive	Positive	Negative
TCGA-D8-A1XM	0	538	57	Positive	Positive	Negative
TCGA-LL-A6FQ	0	80	77	Positive	Positive	Negative
TCGA-BH-A18P	1	921	60	Positive	Negative	Positive
TCGA-E2-A1B4	1	1004	74	Positive	Positive	Negative
TCGA-BH-A0E2	0	435	49	Positive	Positive	Negative
TCGA-A2-A0ER	0	2263	63	Positive	Positive	Equivocal
TCGA-E9-A1RD	0	20	67	NA	NA	NA
TCGA-A2-A04N	0	3188	66	Positive	Positive	NA
TCGA-AQ-A04H	0	754	61	Positive	Positive	NA
TCGA-AO-A12C	0	1994	42	Positive	Positive	Positive
TCGA-BH-A0DT	0	1170	41	Positive	Positive	Negative
TCGA-S3-AA17	0	119	64	Positive	Positive	Equivocal
TCGA-BH-A0BS	0	1641	55	Positive	Positive	[NotAvailable]
TCGA-AR-A5QP	0	622	54	Positive	Positive	Equivocal
TCGA-C8-A1HN	0	99	56	Positive	Positive	Equivocal
TCGA-A2-A1G4	0	595	71	Positive	Positive	Negative
TCGA-D8-A1JD	0	552	41	Positive	Positive	Negative
TCGA-E2-A1LI	0	3121	57	Negative	Negative	Equivocal
TCGA-E2-A14N	0	1434	37	Negative	Negative	Negative
TCGA-AN-A041	0	7	29	Positive	Negative	Positive
TCGA-EW-A1IX	0	1208	48	Positive	Positive	Negative
TCGA-A2-A0CM	1	754	40	Negative	Negative	Negative
TCGA-A8-A09W	0	30	70	Positive	Positive	Negative
TCGA-D8-A1JF	0	366	79	Negative	Negative	Negative

TCGA-AN-A049	0	19	62	Positive	Positive	Negative
TCGA-A2-A1FW	0	528	62	Positive	Negative	Negative
TCGA-AN-A0AL	0	198	41	Negative	Negative	Negative
TCGA-AQ-A54O	0	1001	51	Positive	Positive	Negative
TCGA-BH-A1ES	1	3462	35	Positive	Positive	Negative
TCGA-AC-A6IW	0	413	73	Negative	Negative	Negative
TCGA-AN-A0XS	0	10	63	Negative	Positive	Negative
TCGA-C8-A12M	0	358	70	Positive	Negative	Negative
TCGA-EW-A1OZ	0	1229	56	Positive	Negative	Equivocal
TCGA-A2-A0YF	0	469	67	Positive	Negative	Negative
TCGA-A2-A0T2	1	255	66	Negative	Negative	Negative
TCGA-EW-A1P6	0	562	64	Positive	Positive	Negative
TCGA-3C-AALK	0	1217	52	Positive	Positive	Positive
TCGA-AR-A254	0	2213	50	Positive	Positive	Positive
TCGA-AR-A256	1	2854	45	Negative	Negative	Negative
TCGA-C8-A12Q	1	385	78	Negative	Negative	Positive
TCGA-B6-A40B	0	3152	76	Positive	Positive	Negative
TCGA-B6-A0WZ	0	3941	50	Positive	Positive	NA
TCGA-B6-A0RT	0	2721	39	Negative	Negative	NA
TCGA-BH-A0DD	0	1394	58	Positive	Positive	Positive
TCGA-A8-A097	0	365	65	Positive	Positive	Positive
TCGA-OK-A5Q2	0	64	59	Positive	Positive	Negative
TCGA-A7-A6VW	0	285	48	Negative	Negative	Negative
TCGA-BH-A18H	0	0	63	Positive	Positive	Positive
TCGA-D8-A1XW	0	385	53	Negative	Positive	Negative
TCGA-BH-A0HA	0	1611	31	Positive	Positive	[NotAvailable]
TCGA-PL-A8LZ	0	120	29	NA	NA	NA
TCGA-C8-A26V	0	9	47	Positive	Positive	Negative
TCGA-AN-A0AT	0	10	62	Negative	Negative	Negative
TCGA-HN-A2OB	0	1883	45	Positive	Positive	Negative
TCGA-E2-A150	0	773	48	Negative	Negative	Negative
TCGA-3C-AALJ	0	1228	62	Positive	Positive	NA
TCGA-E2-A10E	0	865	64	Positive	Positive	Equivocal
TCGA-E9-A22E	0	392	56	Positive	Positive	Positive
TCGA-A7-A6VX	0	317	68	Positive	Positive	Negative
TCGA-AN-A03X	0	10	74	Positive	Positive	Positive
TCGA-AR-A24L	1	2866	26	Positive	Positive	Equivocal
TCGA-AO-A0JA	0	655	36	Positive	Positive	Negative
TCGA-D8-A1XO	0	473	56	Positive	Positive	Negative
TCGA-AC-A6IV	0	568	47	Positive	Positive	Equivocal
TCGA-E9-A1RF	0	200	68	NA	NA	NA
TCGA-AO-A0JJ	0	1887	54	Positive	Positive	Negative
TCGA-A8-A084	0	458	81	Positive	Negative	Negative
TCGA-E9-A1N5	0	1120	45	Positive	Positive	Positive

TCGA-C8-A274	0	290	63	Positive	Positive	Negative
TCGA-A2-A0T1	0	521	55	Negative	Negative	Positive
TCGA-BH-A0DE	0	948	62	Positive	Positive	Negative
TCGA-AN-A04A	0	90	36	Positive	Positive	Negative
TCGA-BH-A6R8	0	293	46	Positive	Positive	NA
TCGA-BH-A0E1	0	477	52	Positive	Positive	Equivocal
TCGA-A2-A0T6	0	575	50	Positive	Positive	Negative
TCGA-D8-A27L	0	499	49	Positive	Positive	Negative
TCGA-B6-A0I5	0	4550	49	Positive	Positive	NA
TCGA-AO-A1KO	0	448	46	Positive	Positive	Negative
TCGA-BH-A0HU	0	392	52	Positive	Positive	Negative
TCGA-A2-A25F	0	322	66	Negative	Positive	Negative
TCGA-D8-A1XZ	0	466	81	Positive	Negative	Negative
TCGA-D8-A4Z1	0	659	68	Positive	Positive	Negative
TCGA-A2-A0YK	0	588	61	Positive	Negative	Negative
TCGA-BH-A0BM	0	1876	54	Positive	Negative	Negative
TCGA-AR-A0TY	1	1699	54	Positive	Negative	Negative
TCGA-D8-A1JA	0	502	60	Negative	Negative	Positive
TCGA-BH-A0W7	0	1363	49	Positive	Positive	Negative
TCGA-AC-A3OD	0	451	68	Positive	Positive	Negative
TCGA-AO-A12D	0	1948	43	Negative	Negative	Positive
TCGA-D8-A1J9	0	532	48	Positive	Negative	Positive
TCGA-A8-A07J	0	365	35	Positive	Positive	Negative
TCGA-BH-A0HY	0	1545	60	Positive	Negative	Positive
TCGA-3C-AALI	0	3801	50	Positive	Positive	Positive
TCGA-AC-A2BM	0	1162	41	Positive	Positive	NA
TCGA-A2-A1FX	0	1119	61	Positive	Positive	NA
TCGA-AO-A0J4	0	1587	41	Negative	Negative	Negative
TCGA-EW-A2FV	0	788	39	Positive	Positive	Negative
TCGA-A7-A3RF	0	408	79	Positive	Positive	Negative
TCGA-E2-A15C	0	694	61	Positive	Positive	Equivocal
TCGA-LL-A7SZ	0	170	49	Positive	Positive	Negative
TCGA-EW-A1PC	0	187	66	Positive	Positive	Negative
TCGA-E9-A247	0	828	59	NA	NA	NA
TCGA-E2-A15G	0	554	76	Positive	Positive	Negative
TCGA-AR-A0TU	0	360	35	Negative	Negative	Negative
TCGA-AR-A1AW	0	1935	65	Positive	Positive	Equivocal
TCGA-A2-A0SW	1	1365	82	Positive	Negative	NA
TCGA-S3-A6ZF	0	502	64	Positive	Positive	Equivocal
TCGA-B6-A2IU	0	5176	62	Positive	Positive	NA
TCGA-A7-A26J	0	627	49	Positive	Positive	Negative
TCGA-A7-A425	0	447	70	Positive	Positive	NA
TCGA-BH-A18T	1	224	70	Negative	Negative	Positive
TCGA-GM-A3NW	0	3361	63	Negative	Positive	Equivocal

TCGA-A2-A0EV	0	968	80	Positive	Positive	Negative
TCGA-AR-A24M	0	2947	38	Positive	Positive	Negative
TCGA-E9-A1NF	1	1072	60	Positive	Positive	Negative
TCGA-E2-A15M	1	336	66	Positive	Positive	Negative
TCGA-A1-A0SM	0	242	77	Positive	Negative	Positive
TCGA-A2-A3XZ	0	973	46	Negative	Negative	Positive
TCGA-GM-A2DD	0	1309	53	Negative	Negative	Equivocal
TCGA-A8-A08S	0	1004	71	Positive	Positive	Positive
TCGA-EW-A2FW	0	672	52	Positive	Positive	Negative
TCGA-E9-A22H	0	630	42	Positive	Positive	Positive
TCGA-BH-A0BZ	0	1492	59	Positive	Positive	Negative
TCGA-A2-A3XX	1	1439	49	Negative	Negative	Negative
TCGA-D8-A141	0	626	40	Positive	Positive	Negative
TCGA-BH-A0BQ	0	827	39	Positive	Positive	Positive
TCGA-D8-A1JG	0	366	62	Negative	Negative	Equivocal
TCGA-AQ-A0Y5	1	172	70	Positive	Positive	Positive
TCGA-A8-A06O	0	396	60	Positive	Positive	Negative
TCGA-A8-A09B	0	365	58	Positive	Positive	Negative
TCGA-A2-A0ST	0	3017	62	Negative	Negative	NA
TCGA-BH-A0B8	0	1569	64	Positive	Positive	Equivocal
TCGA-AC-A3W5	0	504	65	Positive	Positive	NA
TCGA-WT-AB41	0	1550	55	Positive	Positive	NA
TCGA-AR-A0U1	0	3101	36	Negative	Negative	Negative
**Validation Set**						
TCGA-A2-A0CO	1	3492	85	Positive	Positive	Negative
TCGA-AR-A0TW	0	3009	50	Positive	Positive	Equivocal
TCGA-AO-A1KP	0	2513	77	Positive	Positive	Negative
TCGA-AC-A6IX	0	373	49	Positive	Positive	NA
TCGA-AO-A0JF	0	650	68	Positive	Positive	Negative
TCGA-AN-A0AS	0	10	70	Positive	Negative	Negative
TCGA-E2-A1L8	0	1205	52	Positive	Positive	Equivocal
TCGA-BH-A0E6	0	293	69	Negative	Negative	Equivocal
TCGA-D8-A1XJ	0	663	76	Positive	Positive	Positive
TCGA-A7-A26H	0	724	72	Positive	Negative	Equivocal
TCGA-B6-A0RM	1	2373	57	Positive	Positive	NA
TCGA-BH-A42U	0	3324	80	Negative	Negative	Negative
TCGA-BH-A0GZ	0	328	62	Positive	Positive	Equivocal
TCGA-E2-A15D	0	526	47	Positive	Positive	Equivocal
TCGA-A7-A5ZV	0	368	62	Negative	Negative	Equivocal
TCGA-EW-A1IW	0	371	80	Positive	Positive	Positive
TCGA-OL-A5D8	0	973	40	Positive	Positive	NA
TCGA-LD-A74U	0	203	79	Positive	Negative	Negative
TCGA-A7-A4SD	0	441	52	Negative	Negative	Equivocal

TCGA-AR-A1AK	0	1559	70	Positive	Positive	Negative
TCGA-AC-A2FG	0	1125	79	Positive	Negative	NA
TCGA-AR-A1AL	0	1485	60	Positive	Positive	Negative
TCGA-E9-A1N4	0	1000	41	Positive	Positive	Positive
TCGA-AR-A1AN	0	1330	46	Positive	Positive	Negative
TCGA-D8-A142	0	425	74	Negative	Negative	Equivocal
TCGA-AN-A0XN	0	10	68	Negative	Positive	Negative
TCGA-E2-A1LK	1	266	84	Negative	Negative	Equivocal
TCGA-EW-A1J1	0	575	38	Positive	Positive	Negative
TCGA-A8-A06Q	0	31	63	Positive	Positive	Negative
TCGA-A8-A09N	0	31	57	Positive	Positive	Positive
TCGA-BH-A1FM	1	1388	44	Positive	Negative	NA
TCGA-AC-A2FO	0	1542	65	Positive	Negative	Negative
TCGA-EW-A1PD	0	424	61	Positive	Positive	Equivocal
TCGA-OL-A5RY	0	752	52	Positive	Negative	NA
TCGA-BH-A0DQ	0	98	42	Positive	Positive	Negative
TCGA-AR-A24U	0	1623	47	Negative	Negative	Positive
TCGA-D8-A1JC	0	480	59	Positive	Positive	Negative
TCGA-A2-A04R	0	2499	36	Positive	Positive	Equivocal
TCGA-C8-A135	0	32	64	Negative	Negative	Positive
TCGA-A2-A0ES	0	1127	52	Positive	Positive	Negative
TCGA-A2-A04Y	0	1099	53	Positive	Positive	Negative
TCGA-D8-A27N	0	146	36	Positive	Positive	Positive
TCGA-A2-A4S0	0	706	77	Positive	Positive	Negative
TCGA-LL-A5YL	0	519	64	Positive	Negative	Positive
TCGA-AN-A0FN	0	218	61	Positive	Positive	Positive
TCGA-A7-A6VY	0	266	48	Negative	Negative	Negative
TCGA-AN-A0AR	0	10	55	Negative	Negative	Negative
TCGA-A8-A06T	0	1614	75	Positive	Positive	Positive
TCGA-E2-A154	0	591	68	Positive	Positive	Negative
TCGA-A8-A08L	1	304	89	Positive	Negative	Negative
TCGA-BH-A0HQ	0	1121	56	Positive	Positive	Negative
TCGA-C8-A273	0	294	29	Positive	Positive	Negative
TCGA-B6-A1KN	0	4233	57	Negative	Negative	NA
TCGA-AN-A0XV	0	162	67	Positive	Positive	Positive
TCGA-BH-A0H3	0	1149	46	Positive	Positive	[NotAvailable]
TCGA-AR-A24O	0	2561	43	Positive	Positive	Negative
TCGA-B6-A0X0	1	3945	54	Positive	Positive	NA
TCGA-AQ-A54N	0	78	51	Negative	Negative	Equivocal
TCGA-A8-A079	0	274	69	Positive	Positive	Negative
TCGA-E2-A107	0	1047	54	Positive	Negative	Equivocal
TCGA-BH-A18N	1	1148	88	Positive	Positive	Negative
TCGA-E2-A14Q	0	1163	50	Positive	Positive	Negative
TCGA-A2-A4S2	0	643	62	Positive	Positive	Negative

TCGA-S3-AA15	0	209	51	Negative	Negative	Negative
TCGA-D8-A27M	0	410	59	Negative	Negative	Negative
TCGA-BH-A0EI	0	743	51	Positive	Positive	Negative
TCGA-E2-A9RU	0	280	90	Positive	Negative	Equivocal
TCGA-BH-A0AU	0	746	45	Positive	Positive	Positive
TCGA-BH-A0BL	0	1340	35	Negative	Negative	Negative
TCGA-E9-A1N3	0	0	70	NA	NA	NA
TCGA-D8-A1X5	0	565	81	Positive	Positive	Positive
TCGA-E2-A1B6	0	867	44	Negative	Negative	Equivocal
TCGA-A8-A07B	0	1308	69	Positive	Positive	Positive
TCGA-A2-A0YH	0	659	53	Positive	Positive	Negative
TCGA-A2-A0T7	0	631	51	Positive	Positive	Negative
TCGA-A8-A08A	0	30	89	Positive	Positive	Negative
TCGA-A2-A0EN	0	2718	70	Positive	Positive	Equivocal
TCGA-A8-A09R	0	273	82	Positive	Positive	Negative
TCGA-C8-A278	0	289	61	Negative	Negative	Positive
TCGA-OL-A6VR	0	1220	48	Positive	Positive	Negative
TCGA-AN-A0FF	0	141	32	Positive	Positive	Negative
TCGA-A1-A0SP	0	584	40	Negative	Negative	Negative
TCGA-E2-A14W	0	974	78	Positive	Positive	NA
TCGA-B6-A0IE	1	1999	38	Negative	Negative	NA
TCGA-E2-A1LA	0	748	59	Positive	Positive	Equivocal
TCGA-D8-A1JM	0	590	59	Positive	Negative	Negative
TCGA-AO-A129	0	2923	29	Negative	Negative	Negative
TCGA-BH-A0AY	0	777	62	Positive	Positive	Negative
TCGA-E2-A1IO	0	638	37	Positive	Positive	Equivocal
TCGA-A1-A0SO	0	852	67	Negative	Negative	Equivocal
TCGA-D8-A1XA	0	839	64	Positive	Positive	Equivocal
TCGA-BH-A1EW	1	1694	38	Negative	Negative	Negative
TCGA-4H-AAAK	0	348	50	Positive	Positive	Equivocal
TCGA-E9-A1RA	0	976	48	NA	NA	NA
TCGA-JL-A3YX	0	352	46	Positive	Positive	Positive
TCGA-D8-A27H	0	397	72	Negative	Negative	Negative
TCGA-E9-A5FK	0	447	60	Positive	Positive	Negative
TCGA-HN-A2NL	0	79	56	Negative	Negative	Negative
TCGA-LL-A6FR	0	489	50	Negative	Positive	Equivocal
TCGA-A2-A25B	0	496	39	Positive	Positive	Equivocal
TCGA-EW-A1J3	0	504	61	Positive	Positive	Positive
TCGA-A8-A09D	0	1522	47	Positive	Positive	Negative
TCGA-E2-A15P	0	595	61	Positive	Positive	Negative
TCGA-BH-A18G	0	61	81	Negative	Negative	Negative
TCGA-EW-A1PG	0	1051	53	Positive	Positive	Equivocal
TCGA-B6-A0RV	0	4788	42	Positive	Positive	NA
TCGA-C8-A132	0	57	56	Positive	Positive	NA

TCGA-GM-A5PV	0	412	63	Positive	Positive	NA
TCGA-B6-A0WT	0	5739	61	Positive	Positive	NA
TCGA-E9-A6HE	0	468	45	Positive	Positive	Negative
TCGA-BH-A0EB	0	745	69	Positive	Positive	Equivocal
TCGA-A2-A259	0	1596	70	Positive	Positive	Negative
TCGA-AR-A0TR	1	160	68	Positive	Positive	Equivocal
TCGA-A2-A0CP	0	2813	60	Positive	Positive	NA
TCGA-A8-A08T	1	3409	64	Positive	Positive	Positive
TCGA-AN-A0FV	0	10	58	Negative	Negative	Positive
TCGA-D8-A1XY	0	503	74	Positive	Positive	Positive
TCGA-C8-A131	0	68	82	Negative	Negative	Negative
TCGA-B6-A0I9	1	369	62	NA	Positive	NA
TCGA-E2-A1BC	0	501	63	Positive	Positive	Negative
TCGA-A2-A25A	0	3276	44	Positive	Positive	Equivocal
TCGA-AR-A0U2	1	2551	47	Positive	Positive	Negative
TCGA-BH-A0HN	0	516	67	Positive	Positive	Equivocal
TCGA-OL-A66O	0	528	39	Positive	Positive	NA
TCGA-C8-A12T	0	0	43	Positive	Positive	Positive
TCGA-E9-A1R2	0	19	51	Positive	Negative	Equivocal
TCGA-E2-A1BD	0	520	53	Positive	Positive	Equivocal
TCGA-A7-A26E	0	954	71	Positive	Positive	Negative
TCGA-BH-A42V	0	272	41	Positive	Positive	Negative
TCGA-AC-A62V	1	348	58	Positive	Positive	NA
TCGA-A8-A07P	0	334	68	Positive	Positive	Positive
TCGA-D8-A1JH	0	426	56	Positive	Positive	Negative
TCGA-A8-A08C	0	881	65	Positive	Positive	Positive
TCGA-B6-A0IB	1	3941	64	Positive	Positive	NA
TCGA-E2-A15A	0	710	45	Positive	Positive	Negative
TCGA-AR-A0TS	0	2275	46	Negative	Negative	Negative
TCGA-LL-A441	0	348	62	Negative	Negative	Negative
TCGA-A7-A56D	0	448	84	Positive	Positive	Negative
TCGA-BH-A0B5	0	1471	40	Positive	Positive	Negative
TCGA-BH-A0BJ	0	660	41	Positive	Positive	Negative
TCGA-BH-A0WA	0	701	82	Negative	Negative	Negative
TCGA-D8-A1XD	0	522	36	Positive	Positive	Negative
TCGA-BH-A1EN	1	2053	78	Negative	Negative	Positive
TCGA-S3-AA10	0	468	65	Negative	Negative	Negative
TCGA-AO-A0JD	0	2190	59	Positive	Positive	Negative
TCGA-LL-A5YN	0	447	46	Positive	Positive	Equivocal
TCGA-C8-A27A	0	371	48	Positive	Positive	Negative
TCGA-A7-A13D	0	965	46	Negative	Positive	Equivocal
TCGA-BH-A0BG	0	756	73	Negative	Negative	Negative
TCGA-EW-A1J5	0	477	59	Positive	Positive	Negative
TCGA-B6-A0WY	1	3461	40	Positive	Negative	NA

TCGA-AR-A2LQ	0	1233	59	Positive	Positive	Negative
TCGA-A2-A0YI	0	441	62	Positive	Positive	Negative
TCGA-A2-A0EY	0	735	62	Positive	Negative	Positive
TCGA-AR-A1AS	0	1150	54	Positive	Positive	Negative
TCGA-E2-A15R	0	519	64	Positive	Positive	Equivocal
TCGA-OL-A5RX	0	513	51	Positive	Positive	NA
TCGA-BH-A0H9	0	1247	69	Positive	Positive	Equivocal
TCGA-E9-A3HO	0	793	49	NA	NA	NA
TCGA-A7-A26G	0	722	50	Negative	Negative	Negative
TCGA-A1-A0SN	0	1196	50	Positive	Positive	Positive
TCGA-EW-A1OX	0	911	43	Positive	Positive	Negative
TCGA-E2-A1AZ	0	2329	63	Negative	Negative	NA
TCGA-A2-A3XT	0	2525	45	Negative	Negative	Negative
TCGA-A8-A07W	0	304	76	Positive	Positive	Negative
TCGA-A7-A0DC	0	906	63	Positive	Negative	Negative
TCGA-E2-A153	0	707	51	Positive	Positive	Negative
TCGA-E2-A2P5	0	515	78	Positive	Positive	NA
TCGA-BH-A18L	1	811	50	Positive	Positive	Negative
TCGA-BH-A1FO	1	785	80	Negative	NA	Negative
TCGA-GM-A2DH	0	1286	58	Negative	Negative	Negative
TCGA-A8-A09A	0	304	40	Positive	Positive	Negative
TCGA-BH-A0EO	0	134	38	Negative	Negative	Negative
TCGA-PE-A5DD	0	1953	64	Positive	Negative	Positive
TCGA-E2-A1II	0	1025	51	Negative	Positive	Negative
TCGA-AC-A2FK	0	1180	45	Positive	Positive	NA
TCGA-A8-A08F	0	1004	59	Positive	Positive	Negative
TCGA-D8-A27I	0	439	58	Positive	Positive	Negative
TCGA-AN-A0FZ	0	10	45	Positive	Negative	Positive
TCGA-BH-A0DZ	0	495	43	Positive	Positive	Positive
TCGA-D8-A1XU	0	395	56	Positive	Positive	Negative
TCGA-D8-A27E	0	530	66	Positive	Positive	Negative
TCGA-E2-A14U	0	1318	74	Positive	Positive	Equivocal
TCGA-A8-A083	0	0	67	Positive	Positive	Negative
TCGA-AN-A0FW	0	11	67	Positive	NA	Negative
TCGA-E9-A1R6	0	84	63	NA	NA	NA
TCGA-AR-A0TZ	1	3262	43	Positive	Positive	Equivocal
TCGA-XX-A899	0	292	46	Positive	Positive	Negative
TCGA-E9-A228	0	381	58	NA	NA	NA
TCGA-E2-A15K	0	275	58	Positive	Positive	Equivocal
TCGA-C8-A1HL	0	317	38	Positive	Negative	Equivocal
TCGA-E2-A158	0	450	43	Negative	Negative	Negative
TCGA-AN-A04D	0	52	58	Negative	Negative	Negative
TCGA-D8-A1JE	0	575	62	Positive	Positive	Negative
TCGA-E2-A1IF	0	1138	74	Positive	Positive	Negative

TCGA-AC-A2QH	0	31	58	Negative	Negative	Negative
TCGA-A7-A426	0	364	50	Positive	Positive	Negative
TCGA-JL-A3YW	0	360	49	Positive	Positive	Positive
TCGA-E2-A573	0	1062	48	Negative	Negative	Equivocal
TCGA-A2-A0EQ	0	2426	64	Negative	Negative	Positive
TCGA-BH-A0HP	0	414	65	Positive	Negative	Negative
TCGA-AC-A3YJ	0	754	66	Positive	Positive	[NotAvailable]
TCGA-B6-A0IQ	0	4285	40	Negative	Negative	NA
TCGA-EW-A1PB	0	608	70	Negative	Negative	Negative
TCGA-A2-A3XY	0	1064	49	Negative	Negative	Negative
TCGA-E2-A56Z	0	219	69	Positive	Positive	Equivocal
TCGA-BH-A1EU	1	1286	83	Positive	Positive	Negative
TCGA-EW-A3E8	0	1035	60	Positive	Negative	Negative
TCGA-EW-A1PF	0	439	50	Positive	Positive	Negative
TCGA-B6-A0IM	0	3836	75	Positive	Positive	NA
TCGA-E2-A14X	0	972	55	Negative	Negative	Negative
TCGA-D8-A1XS	0	496	48	Positive	Positive	Positive
TCGA-AR-A0TT	0	2636	53	Positive	Negative	Negative
TCGA-A8-A07L	0	518	58	Positive	Positive	Negative
TCGA-A2-A0ET	0	1066	58	Positive	Positive	Equivocal
TCGA-BH-A0HX	0	829	54	Positive	Positive	Negative
TCGA-BH-A0AV	0	1180	52	Negative	Negative	[NotAvailable]
TCGA-D8-A1XK	0	441	55	Negative	Negative	Negative
TCGA-D8-A73W	0	244	79	Positive	Negative	Negative
TCGA-AC-A2QJ	0	69	48	Negative	Negative	Negative
TCGA-A1-A0SI	0	635	52	Positive	Positive	Negative
TCGA-A2-A0YJ	0	566	39	Positive	Negative	Negative
TCGA-D8-A1XB	0	552	62	Positive	Positive	Negative
TCGA-C8-A12V	0	385	55	Negative	Negative	Negative
TCGA-C8-A12W	0	385	49	Positive	Positive	Negative
TCGA-D8-A1Y1	1	302	80	Positive	Positive	Negative
TCGA-A2-A4RW	0	222	49	Positive	Positive	Negative
TCGA-A2-A0T3	0	569	37	Positive	Positive	Equivocal
TCGA-D8-A1X6	0	541	80	Positive	Positive	Negative
TCGA-OL-A66K	1	1275	72	Positive	Positive	Equivocal
TCGA-B6-A0IN	1	2573	45	Positive	Negative	NA
TCGA-BH-A0DG	0	713	30	Positive	Negative	Negative
TCGA-D8-A1XQ	0	499	69	Negative	Negative	Negative
TCGA-GM-A5PX	0	551	65	Positive	Positive	Negative
TCGA-A2-A0YG	0	666	63	Positive	Positive	Positive
TCGA-A2-A0D2	0	1027	45	Negative	Negative	Negative
TCGA-E2-A14R	0	1174	62	Negative	Negative	Negative
TCGA-A2-A0CW	0	1948	67	Positive	Positive	Equivocal
TCGA-AQ-A04J	0	819	45	Negative	Negative	Negative

TCGA-A8-A080	0	943	45	Positive	Positive	Negative
TCGA-E2-A1B0	0	1631	50	Negative	Negative	Positive
TCGA-E2-A1LE	1	879	71	Negative	Negative	Positive
TCGA-OL-A5RW	0	1106	40	Negative	Negative	NA
TCGA-B6-A401	0	2596	47	Positive	Positive	Negative
TCGA-E2-A15J	0	590	51	Positive	Positive	Equivocal
TCGA-E2-A15E	0	630	40	Positive	Positive	NA
TCGA-BH-A1FR	1	4398	73	Positive	Positive	NA
TCGA-AC-A2FF	0	1686	40	Positive	Positive	NA
TCGA-AC-A8OR	0	40	75	Positive	Positive	Negative
TCGA-B6-A1KF	0	3088	68	Negative	Negative	Equivocal
TCGA-BH-A18J	1	612	56	Positive	Positive	Negative
TCGA-A2-A0D1	0	1051	76	Negative	Negative	Positive
TCGA-D8-A27T	0	398	53	Positive	Positive	Negative
TCGA-BH-A18Q	1	1692	56	Negative	Negative	Positive
TCGA-LL-A7T0	0	376	70	Positive	Positive	Positive
TCGA-A8-A06Z	0	31	84	Positive	Positive	Negative
TCGA-EW-A1J2	0	403	50	Positive	Positive	Negative
TCGA-E2-A106	0	1807	34	Positive	Positive	Equivocal
TCGA-AR-A1AJ	0	1605	83	Positive	Negative	Negative
TCGA-A1-A0SJ	0	416	39	Positive	Positive	Equivocal
TCGA-LL-A740	0	441	61	Negative	Negative	Equivocal
TCGA-D8-A1JB	0	569	54	Positive	Positive	Equivocal
TCGA-5T-A9QA	0	12	52	Positive	Negative	Equivocal
TCGA-LL-A6FP	0	677	90	Positive	Positive	Equivocal
TCGA-E9-A1RE	0	1054	74	NA	NA	NA
TCGA-A8-A08P	0	943	70	Positive	Positive	Positive
TCGA-LL-A442	0	333	56	Positive	Positive	Equivocal
TCGA-AR-A24N	0	2388	54	Positive	Positive	Equivocal
TCGA-A2-A3Y0	0	1167	57	Positive	Negative	Negative
TCGA-A2-A3XV	0	996	46	Positive	Negative	Equivocal
TCGA-E2-A1B5	0	984	46	Positive	Positive	Negative
TCGA-LL-A9Q3	0	224	69	Positive	Positive	Positive
TCGA-A8-A07R	0	273	80	Negative	Negative	Positive
TCGA-BH-A0HI	0	620	78	Positive	Positive	Negative
TCGA-AO-A12B	0	2359	63	Positive	Positive	Negative
TCGA-A2-A04X	0	1686	34	Positive	Positive	Positive
TCGA-C8-A12Y	0	0	44	NA	NA	NA
TCGA-E9-A1RI	0	1084	43	NA	NA	NA
TCGA-C8-A12U	0	385	46	Positive	Positive	Negative
TCGA-BH-A0B1	0	1148	66	Positive	Positive	Negative
TCGA-E9-A22G	0	715	47	Negative	Negative	Positive
TCGA-E2-A152	0	825	56	Positive	Negative	Positive
TCGA-PL-A8LV	0	7	54	NA	NA	NA

TCGA-A2-A0CK	0	4159	60	Positive	Positive	Negative
TCGA-BH-A1FU	1	2192	44	Negative	Negative	NA
TCGA-E2-A15H	0	393	38	Positive	Positive	Equivocal
TCGA-A8-A099	0	304	76	Positive	Positive	Positive
TCGA-OL-A5D7	0	1416	70	Negative	Negative	NA
TCGA-AR-A1AO	0	1325	47	Positive	Negative	Negative
TCGA-AN-A0FK	0	213	88	Positive	Positive	Positive
TCGA-AO-A12H	0	863	69	Positive	Positive	Negative
TCGA-A2-A0D3	0	875	42	Positive	Positive	Negative
TCGA-E9-A3Q9	0	560	78	Positive	Positive	NA
TCGA-A2-A0YT	1	723	56	Positive	Negative	NA
TCGA-OL-A66H	0	812	74	Positive	Positive	NA
TCGA-BH-A0C3	0	1464	47	Positive	Negative	Negative
TCGA-E9-A5UO	0	420	41	NA	NA	NA
TCGA-A8-A09Q	0	761	83	Positive	Positive	Negative
TCGA-E9-A227	0	24	42	Positive	Positive	Negative
TCGA-A1-A0SQ	0	554	45	Positive	Positive	Negative
TCGA-E9-A229	0	405	37	NA	NA	NA
TCGA-A8-A075	0	518	42	Positive	Positive	Positive
TCGA-A7-A4SA	0	454	40	Positive	Negative	Negative
TCGA-AO-A03N	0	2031	59	Positive	Positive	Negative
TCGA-A2-A0CZ	0	1616	46	Positive	Positive	Negative
TCGA-BH-A0C7	0	1305	48	Positive	Negative	NA
TCGA-BH-A0DS	0	78	71	Positive	Positive	Negative
TCGA-AN-A0FX	0	10	52	Negative	Negative	Positive
TCGA-E9-A1RC	0	1224	56	NA	NA	NA
TCGA-AR-A1AP	0	1216	80	Positive	Positive	Equivocal
TCGA-D8-A1Y0	0	472	65	Positive	Positive	Negative
TCGA-BH-A0GY	0	923	67	Positive	Positive	Negative
TCGA-A8-A092	0	942	48	Positive	Positive	Negative
TCGA-E2-A3DX	0	478	43	Positive	Positive	NA
TCGA-B6-A0I1	1	2361	73	Negative	Negative	NA
TCGA-B6-A0IH	1	3418	81	Positive	Positive	NA
TCGA-E2-A15S	0	428	34	Positive	Negative	Equivocal
TCGA-E2-A10A	0	1229	41	Positive	Positive	Positive
TCGA-AC-A2B8	0	45	84	Positive	Positive	Negative
TCGA-GM-A3NY	0	1162	72	Positive	Positive	Negative
TCGA-A2-A0CX	0	1728	52	Positive	Negative	Positive
TCGA-AO-A03M	0	1866	29	Positive	Positive	Negative
TCGA-AO-A12E	0	1743	51	Positive	Positive	Negative
TCGA-AR-A252	0	2226	50	Positive	Positive	Negative
TCGA-AR-A24Z	0	2403	57	Positive	Positive	Negative
TCGA-D8-A1XF	0	463	45	Positive	Positive	Negative
TCGA-A7-A0DB	0	1007	56	Positive	Positive	Negative

TCGA-B6-A0X5	1	2097	61	Positive	Positive	NA
TCGA-BH-A8G0	0	662	54	Positive	Positive	Negative
TCGA-A2-A3KD	0	1206	47	Positive	Positive	Negative
TCGA-PL-A8LY	0	8	30	NA	NA	NA
TCGA-BH-A28Q	0	196	46	Positive	Positive	Negative
TCGA-AN-A0FD	0	196	71	Positive	Positive	Positive
TCGA-A2-A25D	0	552	90	Positive	Negative	Negative
TCGA-A7-A13F	0	765	44	Positive	Positive	Equivocal
TCGA-E2-A108	0	837	64	Positive	Positive	Negative
TCGA-E2-A14V	0	1042	53	Positive	Positive	Positive
TCGA-BH-A18R	1	1142	50	NA	Negative	Positive
TCGA-AR-A1AH	0	2298	51	Positive	Negative	Negative
TCGA-AR-A24T	0	2312	46	Positive	Positive	Equivocal
TCGA-AC-A5XS	0	47	74	Positive	Positive	Negative
TCGA-C8-A26W	0	7	58	Positive	Positive	Equivocal
TCGA-BH-A18U	1	1563	72	Positive	Positive	Positive
TCGA-B6-A0IG	1	4456	50	Positive	Positive	NA
TCGA-AN-A0FT	0	183	63	Positive	Positive	Positive
TCGA-E2-A15O	0	396	89	Positive	Positive	Equivocal
TCGA-B6-A0IO	0	3350	66	Positive	NA	NA
TCGA-E2-A574	0	605	44	Negative	Negative	Equivocal
TCGA-A1-A0SK	1	967	54	Negative	Negative	Negative
TCGA-E2-A14Z	1	563	64	Positive	Positive	Negative
TCGA-A8-A06P	0	396	63	Positive	Positive	Negative
TCGA-E2-A1IE	0	1165	61	Positive	Positive	Equivocal
TCGA-D8-A73U	0	492	88	Positive	Positive	Negative
TCGA-AO-A0J5	1	792	48	Positive	Negative	Equivocal
TCGA-E2-A14Y	0	870	35	Positive	Positive	Equivocal
TCGA-E2-A1L9	0	598	40	Positive	Positive	Negative
TCGA-E9-A1N8	0	365	48	Negative	NA	Negative
TCGA-BH-A0C1	0	1339	61	Positive	Positive	Negative
TCGA-A2-A0CV	0	1930	41	Positive	Positive	Negative
TCGA-AR-A1AV	0	1864	68	Positive	Positive	Negative
TCGA-BH-A1FC	1	3472	78	Negative	Negative	Negative
TCGA-AO-A12F	0	1471	36	Negative	Negative	Negative
TCGA-D8-A1JT	0	405	70	Positive	Positive	Equivocal
TCGA-B6-A0IA	0	6719	51	Positive	Positive	NA
TCGA-AC-A2BK	0	1172	78	Negative	Negative	Negative
TCGA-BH-A0HK	0	178	81	Positive	Negative	Negative
TCGA-S3-AA12	0	259	82	Positive	Negative	Negative
TCGA-AN-A0XR	0	10	55	Positive	Negative	Negative
TCGA-A2-A04T	0	2246	62	Negative	Negative	Equivocal
TCGA-AR-A2LN	0	1161	65	Positive	Positive	Negative
TCGA-A7-A6VV	0	313	51	Negative	Negative	Negative

TCGA-A8-A08J	1	1127	52	Positive	Negative	Negative
TCGA-A7-A4SB	0	418	56	Positive	Positive	Negative
TCGA-EW-A3U0	0	532	61	Negative	Negative	Negative
TCGA-C8-A1HG	0	345	50	Positive	Positive	Negative
TCGA-BH-A1FG	1	3738	88	Positive	Positive	Negative
TCGA-AR-A255	0	1792	62	Positive	Positive	Positive
TCGA-A8-A07C	0	1034	57	Negative	Negative	Negative
TCGA-A1-A0SE	0	1321	56	Positive	Positive	Negative
TCGA-BH-A0HB	0	806	55	Positive	Positive	Equivocal
TCGA-A8-A0AB	0	518	54	Positive	Positive	Positive
TCGA-A8-A07F	0	577	65	Positive	Positive	Negative
TCGA-BH-A209	1	3959	77	Positive	Positive	NA
TCGA-A2-A4RY	0	648	46	Positive	Positive	Negative
TCGA-AC-A3TN	0	34	75	Positive	Positive	Positive
TCGA-BH-A1FJ	1	1927	66	Negative	Positive	NA
TCGA-B6-A0RU	0	3991	49	Negative	Negative	NA
TCGA-AC-A3HN	0	21	87	Positive	Positive	Negative
TCGA-BH-A0DV	0	1374	54	Positive	Positive	Negative
TCGA-AO-A0J3	0	651	67	Positive	Positive	Equivocal
TCGA-E2-A109	0	1417	64	Positive	Negative	Equivocal
TCGA-A7-A3IY	0	345	71	Positive	Positive	Negative
TCGA-A8-A08I	0	365	53	Positive	Positive	Negative
TCGA-AO-A03R	0	2091	57	Positive	Positive	Negative
TCGA-E9-A24A	0	747	69	NA	NA	NA
TCGA-B6-A1KI	0	2236	63	Positive	Positive	Equivocal
TCGA-E9-A1NC	0	1203	61	Negative	Positive	Positive
TCGA-AO-A03P	1	2911	54	Positive	Positive	Negative
TCGA-Z7-A8R5	0	3287	61	Positive	Positive	Negative
TCGA-EW-A1P4	0	907	43	Negative	Negative	Negative
TCGA-AO-A0J9	0	1613	61	Positive	Positive	NA
TCGA-D8-A1JP	0	639	73	Positive	Positive	Negative
TCGA-C8-A12O	0	385	50	Positive	Positive	Negative
TCGA-AR-A24X	0	2548	52	Positive	Positive	Equivocal
TCGA-GM-A2DC	0	1628	57	Positive	Positive	NA
TCGA-AR-A1AI	0	1881	47	Negative	Negative	Equivocal
TCGA-AQ-A1H3	0	989	49	Positive	Positive	Negative
TCGA-C8-A12P	0	358	55	Negative	Negative	Positive
TCGA-AR-A1AT	1	1272	62	Positive	Positive	Positive
TCGA-D8-A1XC	1	377	85	Positive	Positive	Negative
TCGA-E2-A15I	0	530	44	Positive	Positive	Equivocal
TCGA-B6-A0RS	1	3063	38	Negative	Negative	NA
TCGA-BH-A0DX	0	1442	62	Positive	Positive	Negative
TCGA-E2-A1B1	0	1613	45	Positive	Positive	Equivocal
TCGA-D8-A1JJ	0	611	54	Positive	Positive	Negative

TCGA-AR-A2LM	0	1218	49	Positive	Positive	NA
TCGA-GM-A3XL	0	2108	49	Negative	Negative	NA
TCGA-BH-A0HF	0	727	77	Positive	Positive	Negative
TCGA-A7-A4SE	0	644	54	Negative	Negative	Negative
TCGA-AO-A1KT	0	541	78	Positive	Positive	Equivocal
TCGA-LL-A73Y	0	126	67	Negative	Negative	Negative
TCGA-D8-A27V	0	381	62	Positive	Positive	Negative
TCGA-LQ-A4E4	0	849	73	Positive	Positive	NA
TCGA-A7-A26I	0	661	65	Negative	Negative	Equivocal
TCGA-D8-A1JS	0	371	77	Positive	Positive	Negative
TCGA-BH-A0BW	0	355	71	Negative	Negative	NA
TCGA-AO-A125	0	3019	72	Positive	Positive	Negative
TCGA-BH-A28O	0	324	50	Positive	Positive	Negative
TCGA-BH-A201	0	210	64	Positive	Positive	Negative
TCGA-BH-A0HL	0	72	56	Positive	Positive	Equivocal
TCGA-A8-A09E	0	1492	73	Positive	Positive	Positive
TCGA-BH-A1F2	1	959	53	Positive	Positive	Positive
TCGA-B6-A1KC	0	1326	67	Positive	Negative	Negative
TCGA-A7-A0CJ	0	931	57	Positive	Positive	Negative
TCGA-B6-A0RE	0	6435	61	Negative	Negative	NA
TCGA-AR-A2LJ	0	1912	40	Positive	Positive	Positive
TCGA-A8-A06R	0	547	69	Positive	Negative	Positive
TCGA-BH-A0B3	0	1203	53	Negative	Negative	Negative
TCGA-Z7-A8R6	0	3256	46	Positive	Positive	Negative
TCGA-BH-A0DK	0	423	49	Positive	Positive	Negative
TCGA-LD-A66U	0	646	44	Positive	Positive	Negative
TCGA-A8-A09K	0	912	68	Positive	Positive	Negative
TCGA-BH-A0BA	0	1132	51	Positive	Positive	Negative
TCGA-A2-A3KC	0	635	55	Positive	Positive	Equivocal
TCGA-AN-A03Y	0	10	66	Positive	Positive	Negative
TCGA-A8-A0A6	0	640	64	Positive	Positive	Negative
TCGA-AC-A3QP	0	675	79	Positive	Positive	Equivocal
TCGA-A2-A0YL	0	1474	48	Positive	Positive	Negative
TCGA-B6-A0RG	0	2082	26	Negative	Negative	NA
TCGA-AR-A5QN	0	488	68	Positive	Positive	Positive
TCGA-BH-A204	1	2534	80	NA	NA	NA
TCGA-AQ-A1H2	0	475	84	Positive	Positive	NA
TCGA-C8-A12K	0	0	80	NA	NA	NA
TCGA-E9-A249	0	29	45	NA	NA	NA
TCGA-BH-A208	1	1759	48	NA	NA	NA
TCGA-A2-A1FZ	0	683	63	Positive	Positive	Negative
TCGA-D8-A1JI	0	577	54	Positive	Positive	Negative
TCGA-GM-A2DA	0	5909	46	Positive	Positive	Equivocal
TCGA-A7-A0CD	0	1165	66	Positive	Positive	Equivocal

TCGA-BH-A0AZ	0	867	47	Positive	Positive	Negative
TCGA-LD-A7W6	0	140	54	Positive	Positive	Negative
TCGA-PE-A5DE	0	2645	41	Positive	Positive	Negative
TCGA-BH-A0W5	0	1288	77	Positive	Positive	Equivocal
TCGA-B6-A0IK	1	571	63	Negative	Negative	NA
TCGA-AO-A03V	0	886	41	Positive	Positive	Negative
TCGA-A2-A0EU	0	1043	79	Positive	Positive	Negative
TCGA-A8-A096	0	0	73	Positive	Positive	Negative
TCGA-A2-A04V	1	1920	39	Positive	Positive	Equivocal
TCGA-D8-A1J8	0	431	77	Positive	Positive	Negative
TCGA-A2-A04U	0	671	47	Negative	Negative	Negative
TCGA-A8-A081	0	0	80	Positive	Positive	Negative
TCGA-D8-A3Z6	0	563	56	Positive	Positive	Negative
TCGA-AN-A0XT	0	10	54	Positive	Negative	Negative
TCGA-D8-A27W	0	373	55	Positive	Positive	Equivocal
TCGA-EW-A1PE	0	320	56	Positive	Positive	Negative
TCGA-AC-A8OP	0	614	72	Positive	Positive	NA
TCGA-AC-A8OQ	0	34	72	Negative	Negative	NA
TCGA-C8-A27B	0	30	48	Negative	Negative	Negative
TCGA-AO-A0JL	0	1683	59	Negative	Negative	Negative
TCGA-E2-A15F	0	658	64	Positive	Positive	Negative
TCGA-BH-A6R9	0	160	61	Negative	Positive	Negative
TCGA-BH-A1FL	1	1673	69	Positive	Positive	NA
TCGA-S3-A6ZH	0	515	29	Positive	Positive	Equivocal
TCGA-AR-A24R	0	2653	45	Positive	Positive	Negative
TCGA-C8-A275	0	1	56	NA	NA	NA
TCGA-AO-A1KS	0	16	69	Positive	Positive	Equivocal
TCGA-BH-A18F	0	268	50	Positive	Positive	Negative
TCGA-BH-A1EY	1	538	79	Positive	Positive	Negative
TCGA-A8-A09Z	0	0	83	Positive	Negative	Negative
TCGA-D8-A1XL	0	606	34	Positive	Positive	Equivocal
TCGA-A2-A04Q	0	1276	48	Negative	Negative	Equivocal
TCGA-AO-A12G	0	1266	75	Positive	Positive	Positive
TCGA-AC-A3W6	0	602	90	Positive	Positive	Negative
TCGA-BH-A1EV	1	365	45	Positive	Positive	Positive
TCGA-D8-A1Y3	0	430	61	Positive	Positive	NA
TCGA-E2-A1LB	0	1231	41	Negative	Negative	Positive
TCGA-B6-A0WS	1	2965	58	Positive	Positive	NA
TCGA-C8-A3M7	0	1	60	Negative	Negative	Negative
TCGA-AR-A0U4	0	2615	54	Negative	Negative	Negative
TCGA-E9-A1NG	1	786	62	Positive	Positive	Negative
TCGA-BH-A203	1	1174	78	NA	NA	NA
TCGA-B6-A0WV	1	2422	67	Positive	Positive	NA
TCGA-EW-A1OW	0	694	58	Negative	Negative	Negative

TCGA-AR-A2LK	0	1304	62	Positive	Positive	Equivocal
TCGA-BH-A0BT	0	1386	56	Positive	Positive	Negative
TCGA-A8-A07U	0	760	66	Negative	Positive	Negative
TCGA-A8-A090	0	0	74	Positive	Positive	Positive
TCGA-D8-A146	0	643	57	Positive	Positive	Negative
TCGA-E9-A248	0	59	51	NA	NA	NA
TCGA-D8-A1JL	0	611	72	Negative	Negative	Negative
TCGA-A1-A0SG	0	434	61	Positive	Positive	Negative
TCGA-A8-A0A9	0	396	80	Positive	Positive	Negative
TCGA-BH-A0E9	0	1405	53	Positive	Positive	Negative
TCGA-AR-A251	0	2387	51	Positive	Negative	Equivocal
TCGA-AO-A0JG	0	798	49	Positive	Positive	Negative
TCGA-AR-A2LL	0	2012	70	Positive	Positive	Equivocal
TCGA-GM-A2DL	0	2763	50	Positive	Positive	Negative
TCGA-AR-A0TV	0	905	66	Positive	Positive	Equivocal
TCGA-AO-A1KR	0	2141	51	Negative	Negative	Negative
TCGA-A2-A0SU	0	1662	66	Positive	Positive	Negative
TCGA-A8-A07Z	0	853	85	Positive	Positive	Negative
TCGA-D8-A3Z5	0	1015	54	Positive	Positive	Negative
TCGA-E2-A1LS	0	470	46	Negative	Negative	Negative
TCGA-AR-A2LO	0	1198	46	Positive	Positive	Equivocal
TCGA-OL-A5DA	0	1783	61	Positive	Positive	NA
TCGA-A8-A09V	0	457	51	Positive	Positive	Negative
TCGA-AN-A0XU	0	10	54	Negative	Negative	Negative
TCGA-EW-A1PO	0	1251	55	Positive	Negative	Negative
TCGA-BH-A8FY	1	295	87	Positive	Positive	Negative
TCGA-AO-A128	0	3248	61	Negative	Negative	Negative
TCGA-B6-A0I8	1	749	46	NA	NA	NA
TCGA-D8-A1XT	0	193	61	Negative	Negative	Positive

a. Overall Survival. b. the age at diagnosis

Supplementary Table S2: Univariate Cox regression analysis reveal significant relation between lncRNA expression and survival time

Gene symbol	Chromosomal position	P-Value
CAT304	chr2:122407202-122409328	2.10E-05
STXBP5-AS1	chr6:146841388-147204614	4.10E-05
C7orf29|113763	chr7:150004597-150035352	9.40E-05
FLJ90757|440465	chr17:78988880-79008646	1.60E-04
FLJ35776|649446	chr18:3495841-3874284	2.60E-04
CAT104	chr1:148879196-148879701	2.80E-04
LOC282997|282997	chr10:112628646-112658585	3.60E-04
LINC01234	chr12:113744577-113773683	6.90E-04

LOC100188949	chr13:30913240-30951282	9.00E-04
CAT1952	chr17:48633027-48639664	1.00E-03
CAT2247	chr22:39056343-39077882	1.00E-03

Supplementary Table S3: Three lncRNAs significantly associated with overall survival of patients in the training-set (n = 532)

Gene name	TCGA Gene_ID[a]	Genomic coordinates	*P*-value	Hazard ratio	Coefficient[b]
CAT104	*uc001emi.3*	chr1:14887919 6- 148879701	2.8E-04	0.771	−0.26
LINC01234	*uc001tvk.1*	chr12:1137445 77- 113773683	6.9E-04	0.771	0.201
STXBP5-AS1	*uc003qlu.1*	chr6:14684138 8..147204614	3.9E-05	1.673	0.514

Supplementary Table S4. Clinical characteristics of breast cancer patients belonging to low or high risk group in the training set, the Validation set and the total patient set

Characteristic	Training set		*P* Value	Validation set		*P* Value	Total set		*P* Value
	Low-risk	High-risk		Low-risk	High-risk		Low-risk	High-risk	
Mean Age	58.85	58.16	0.53[a]	57.41	59.45	0.48[a]	58.20	58.76	0.09[a]
Mean OS_time	1113	982	1.37E-07[a]	1189	888	2.04E-06[a]	1145	942	7.18E-12[a]
Vital Status									
Living	251	218	9.51E-06[b]	245	225	6.80E-04[b]	496	443	4.51E-07[b]
Dead	15	48		21	41		36	89	

a. Log-rank test, b. Chi-square test.

Supplementary Table S5: Kaplan–Meier and ROC analyses of three-lncRNA signature and ten known biomarkers

Biomarker	AUC	*P*-value	95% CI of AUC	*P*-value[a]
Three_lncRNA	0.752	<0.001	0.65-0.85	<0.001
TP53	0.582	0.159	0.47-0.69	0.329
ESR1	0.501	0.991	0.39-0.61	0.251
PGR	0.546	0.427	0.43-0.66	0.235
ERBB2	0.599	0.088	0.48-0.71	0.245
HOTAIR	0.580	0.054	0.47-0.68	0.452
MKI67	0.509	0.877	0.40-0.62	0.71
MAPT	0.645	0.012	0.54-0.75	0.011

SLC7A5	0.529	0.623	0.42-0.64	0.241
GNB2L1	0.606	0.068	0.50-0.72	0.164
RSF1	0.573	0.208	0.46-0.69	0.164
Two-gene *(MMP, LCN2)*	0.511	0.855	0.39-0.63	0.205

a. log-rand test

Supplementary Table S6: Kaplan–Meier and ROC analyses of three-lncRNA signature together with different clinical characteristics

**Biomarker**	**AUC**	***P*-value**	**95% CI of AUC**	***P*-value (log-rand)**
Three_lncRNA	0.752	1.43E-05	0.65-0.85	6.33E-08
Age	0.651	9.01E-03	0.54-0.76	1.10E-02
Three_lncRNA and age	0.752	2.15E-04	0.65-0.85	2.10E-07
Three_lncRNA and stage	0.750	2.36E-05	0.65-0.85	2.15E-04
Three_lncRNA and subtype	0.740	1.24E-05	0.62-0.85	5.20E-04

Supplementary Table S7: KEGG-Pathway enrichment analysis of mRNA targets of miRNAs associated with three lncRNAs

**KEGG_Pathway**	**Count**	**P-Value**	**Benjamini**	**Gene**
Focal adhesion	20	7.90E-04	1.10E-01	*COL4A4, COL4A2, PDGFB, DIAPH1, ITGB4, ACTN1, IGF1, COL2A1, PAK6, ACTG1, LAMA3, ITGA6, ITGB8, COL6A3, PDGFRB, RAP1B, THBS2, COL11A1, PIK3R1, MYLK*
Hypertrophic cardiomyopathy (HCM)	12	8.40E-04	6.10E-02	*TNNT2, ACTG1, ITGA6, ITGB8, LMNA, ITGB4, IGF1, CACNA2D3, CACNA1C, CACNA1D, EMD, TPM3*
Arrhythmogenic right ventricular cardiomyopathy (ARVC)	11	1.30E-03	6.10E-02	*ACTG1, ITGA6, ITGB8, LMNA, ITGB4, ACTN1, CACNA2D3, CACNA1C, CTNNA1, CACNA1D, EMD*
Dilated cardiomyopathy	12	1.60E-03	5.90E-02	*TNNT2, ACTG1, ITGA6, ITGB8, LMNA, ITGB4, IGF1, CACNA2D3, CACNA1C, CACNA1D, EMD, TPM3*

ECM-receptor interaction	11	2.70E-03	7.90E-02	*COL4A4, CD47, COL4A2, LAMA3, ITGA6, ITGB8, COL6A3, ITGB4, COL2A1, THBS2, COL11A1*
Regulation of actin cytoskeleton	19	4.20E-03	1.00E-01	*FGFR2, FGF18, FGF7, PDGFB, DIAPH1, SSH2, ITGB4, ACTN1, PAK6, ACTG1, ITGA6, ITGB8, ARPC5L, PDGFRB, PIK3R1, MYLK, MYH10, SLC9A1, FGF4*
Pathways in cancer	25	5.80E-03	1.20E-01	*COL4A4, DVL2, FGFR2, FGF18, COL4A2, FGF7, PDGFB, NFKBIA, FASLG, IGF1, TFG, SMAD2, KIT, CTNNA1, RALGDS, CDK2, STAT3, TPM3, CCNE2, LAMA3, HIF1A, ITGA6, PDGFRB, PIK3R1, FGF4*
Notch signaling pathway	7	1.40E-02	2.30E-01	*DVL2, NOTCH2, APH1A, DTX2, JAG2, RBPJ, NCOR2*
MAPK signaling pathway	20	1.80E-02	2.60E-01	*FGFR2, FGF18, FGF7, TAOK2, PDGFB, NLK, TAOK3, FASLG, CACNA2D3, MAP4K3, RPS6KA3, RPS6KA4, MAP3K8, PLA2G2A, PDGFRB, RAP1B, CACNA1C, CACNA1D, MAP3K11, FGF4*
Ubiquitin mediated proteolysis	12	3.00E-02	3.70E-01	*UBE2E3, UBE2O, UBE2Z, TRIM32, BIRC6, SIAH1, CDC34, UBE3C, UBE2NL, CUL4B, FBXW11, UBE2E1*
Tight junction	11	5.80E-02	5.60E-01	*ACTG1, MAGI3, TJP1, EPB41, ACTN1, CLDN11, CSDA, CTNNA1, CLDN14, MYH10, SPTAN1*
Small cell lung cancer	8	6.50E-02	5.70E-01	*COL4A4, CCNE2, COL4A2, LAMA3, ITGA6, NFKBIA, CDK2, PIK3R1*
Calcium signaling pathway	13	7.00E-02	5.70E-01	*PTGER3, ERBB3, P2RX4, GNAL, ADRB2, P2RX1, ADRA1B, PDGFRB, CACNA1C, PLCB1, CACNA1D, MYLK, HTR2A*
Melanoma	7	8.00E-02	5.90E-01	*FGF18, FGF7, PDGFB, PDGFRB, IGF1, PIK3R1, FGF4*
Neurotrophin signaling pathway	10	8.00E-02	5.70E-01	*MAGED1, NTRK3, RPS6KA3, RPS6KA4, GAB1, NFKBIA, FASLG, RAP1B, FRS2, PIK3R1*
Prostate cancer	8	8.30E-02	5.60E-01	*CCNE2, FGFR2, PDGFB, NFKBIA, PDGFRB, IGF1, CDK2, PIK3R1*

Supplementary Table S8: The average expression levels and differences of genes in the interaction network and grouped based on lncRNA *CAT104*

Gene	Group with low expressed *CAT104*	Group with high expressed *CAT104*	*P*-Value
CAT104	5.145	7.320	2.30E-235
hsa-mir-3200	2.363	1.723	4.47E-12
hsa-mir-18a	3.311	2.978	5.54E-04
hsa-mir-17	9.169	8.888	3.57E-04
GFRA1	10.337	11.246	1.37E-05
ADRB2	4.931	5.403	5.88E-09
LDB2	7.562	7.919	2.52E-10
ID1	7.376	7.859	5.65E-12
TENC1	10.173	10.534	2.01E-12
COL6A3	13.941	14.378	8.55E-08
CACNA1C	7.441	7.924	1.81E-11
PDGFRB	11.524	11.941	1.19E-11
ADAMTS6	5.474	5.912	4.34E-10
COL7A1	6.966	7.795	3.09E-13
DBNL	10.862	11.034	1.84E-08
SMAD6	6.082	6.346	1.67E-06
CLDN11	6.232	6.758	2.07E-07
ERG	7.852	8.138	8.35E-07
ZFPM2	6.363	6.727	3.33E-06
FGF18	3.263	3.810	6.89E-12
NPR1	6.891	7.244	2.02E-06
NOX4	7.037	7.438	8.41E-08
SLC31A2	8.470	8.750	3.59E-07
TRIM8	10.957	11.151	1.10E-08
ADAMTS7	7.176	7.402	6.11E-04
CDK9	10.007	10.193	9.03E-12
PRDM8	4.120	4.553	2.43E-10

Supplementary Table S9: The average expression levels and differences of genes in the interaction network and grouped based on lncRNA *LINC01234*

Gene	Group with low expressed *LINC01234*	Group with high expressed *LINC01234*	*P*-Value
LINC01234	0.021	1.715	2.20E-101
hsa-mir-190b	3.849	3.085	1.16E-09
IGF2BP2	4.928	5.769	2.25E-12
ABTB2	6.523	7.232	5.39E-16
TBPL1	7.622	7.858	6.73E-11
KPNA2	11.176	11.646	1.11E-15

MTHFD1L	7.881	8.402	7.01E-14
BRD7	10.009	10.206	5.41E-11
ATAD5	6.503	6.98	3.37E-14
CIRH1A	9.423	9.716	7.65E-16
OGDHL	1.835	2.658	1.15E-10
UBE2O	9.951	10.126	4.39E-08
WDR43	9.82	10.071	2.05E-15
CCNB1	9.614	9.99	6.39E-11
CDCA3	7.362	7.837	4.67E-10
SENP5	9.489	9.706	9.76E-15
POLR2D	9.026	9.231	1.52E-13
CENPN	7.923	8.453	4.28E-20
PSAT1	5.762	7.085	8.02E-20
KIF11	9.185	9.607	7.98E-12
SLC30A3	1.648	2.103	1.05E-08
SMAP1	9.447	9.654	5.63E-11

Supplementary Table S10: The average expression levels and differences of genes in the interaction network and grouped based on lncRNA *STXBP5-AS1*

Gene	Group with low expressed *STXBP5-AS1*	Group with high expressed *STXBP5-AS1*	*P*-Value
STXBP5-AS1	0.629	2.141	9.40E-250
hsa-mir-421	1.835	1.699	1.73E-02
hsa-mir-18a	3.253	3.036	2.49E-02
hsa-mir-17	9.116	8.941	2.69E-02
GCNT4	4.114	4.666	5.36E-09
IQSEC1	10.518	10.713	7.79E-09
SYNJ1	8.102	8.471	3.74E-20
ADAMTS6	5.434	5.952	1.19E-13
HIF1A	10.811	11.197	1.58E-09
CACNA1C	7.442	7.923	2.13E-11
FNDC3A	10.104	10.508	3.53E-21
FBXW11	10.067	10.249	6.84E-09
AP3B1	10.383	10.549	1.57E-09
TJP1	10.58	10.914	3.26E-19
SAMD4A	7.747	8.107	2.54E-09
BICC1	5.878	6.955	4.02E-23
PTPRG	8.437	9.18	9.12E-27
PDGFRB	11.475	11.99	4.17E-17
LDB2	7.526	7.956	1.95E-14
ARID4B	10.449	10.695	3.18E-12
APOOL	7.108	7.608	4.71E-16
GNAL	4.892	5.47	3.29E-12
TP53BP1	10.189	10.424	4.10E-10

DOCK4	8.142	8.649	8.02E-21
RANBP2	11.468	11.71	7.71E-12
GJC1	7.534	7.947	4.03E-13
MYLK	10.456	11.041	2.96E-15
AFF1	10.603	10.87	4.84E-09
FGF7	6.826	7.36	1.40E-11
ZFR	10.98	11.191	8.76E-13
ZBTB26	6.29	6.645	1.14E-12
SP3	10.991	11.17	1.33E-08
SENP6	10.552	10.758	5.45E-12
PRTG	5.226	5.79	7.49E-09
STK38L	9.99	10.273	2.95E-08
ERG	7.786	8.204	3.56E-13
MYO5A	9.576	9.965	1.23E-17
ZFPM2	6.267	6.823	8.73E-13
NDST2	9.052	9.209	2.89E-09
PTPRB	8.572	9.178	9.27E-20
TRIP11	9.853	10.159	7.36E-13
ZNF704	7.618	8.137	2.30E-10
STK17B	9.476	9.812	3.23E-10
GAB1	9.32	9.595	5.18E-09
MTM1	8.281	8.546	1.19E-12
COL6A3	13.871	14.448	1.20E-12
RNF111	9.17	9.448	4.46E-15
CUL4B	10.846	11.006	2.00E-09
RPS6KA3	9.152	9.613	4.76E-15
